# Four decades of full-scale nitrous oxide emission inventory in China

**DOI:** 10.1093/nsr/nwad285

**Published:** 2023-11-06

**Authors:** Minqi Liang, Zheyan Zhou, Peiyang Ren, Han Xiao, Xu-Ri, Zhongmin Hu, Shilong Piao, Hanqin Tian, Qing Tong, Feng Zhou, Jing Wei, Wenping Yuan

**Affiliations:** School of Atmospheric Sciences, Guangdong Province Data Center of Terrestrial and Marine Ecosystems Carbon Cycle, Sun Yat-sen University, Zhuhai 510245, China; School of Atmospheric Sciences, Guangdong Province Data Center of Terrestrial and Marine Ecosystems Carbon Cycle, Sun Yat-sen University, Zhuhai 510245, China; School of Atmospheric Sciences, Guangdong Province Data Center of Terrestrial and Marine Ecosystems Carbon Cycle, Sun Yat-sen University, Zhuhai 510245, China; School of Atmospheric Sciences, Guangdong Province Data Center of Terrestrial and Marine Ecosystems Carbon Cycle, Sun Yat-sen University, Zhuhai 510245, China; State Key Laboratory of Tibetan Plateau Earth System, Resources and Environment, Institute of Tibetan Plateau Research, Chinese Academy of Sciences, Beijing 100101, China; Key Laboratory of Agro-Forestry Environmental Processes and Ecological Regulation of Hainan Province, Hainan University, Haikou 570228, China; Sino-French Institute for Earth System Science, College of Urban and Environmental Sciences, Peking University, Beijing 100871, China; Schiller Institute for Integrated Science and Society, Department of Earth and Environmental Sciences, Boston College, Chestnut Hill, MA 02467, USA; Institute of Energy, Environment and Economy, Tsinghua University, Beijing 100084, China; Sino-French Institute for Earth System Science, College of Urban and Environmental Sciences, Peking University, Beijing 100871, China; School of Atmospheric Sciences, Guangdong Province Data Center of Terrestrial and Marine Ecosystems Carbon Cycle, Sun Yat-sen University, Zhuhai 510245, China; Southern Marine Science and Engineering Guangdong Laboratory (Zhuhai), Zhuhai 519082, China; School of Atmospheric Sciences, Guangdong Province Data Center of Terrestrial and Marine Ecosystems Carbon Cycle, Sun Yat-sen University, Zhuhai 510245, China; Southern Marine Science and Engineering Guangdong Laboratory (Zhuhai), Zhuhai 519082, China

**Keywords:** nitrous oxide, four decades, anthropogenic sources, natural sources

## Abstract

China is among the top nitrous oxide (N_2_O)-emitting countries, but existing national inventories do not provide full-scale emissions including both natural and anthropogenic sources. We conducted a four-decade (1980**–**2020) of comprehensive quantification of Chinese N_2_O inventory using empirical emission factor method for anthropogenic sources and two up-to-date process-based models for natural sources. Total N_2_O emissions peaked at 2287.4 (1774.8**–**2799.9) Gg N_2_O yr^−1^ in 2018, and agriculture-developed regions, like the East, Northeast, and Central, were the top N_2_O-emitting regions. Agricultural N_2_O emissions have started to decrease after 2016 due to the decline of nitrogen fertilization applications, while, industrial and energetic sources have been dramatically increasing after 2005. N_2_O emissions from agriculture, industry, energy, and waste represented 49.3%, 26.4%, 17.5%, and 6.7% of the anthropogenic emissions in 2020, respectively, which revealed that it is imperative to prioritize N_2_O emission mitigation in agriculture, industry, and energy. Natural N_2_O sources, dominated by forests, have been steadily growing from 317.3 (290.3**–**344.1) Gg N_2_O yr^−1^ in 1980 to 376.2 (335.5**–**407.2) Gg N_2_O yr^−1^ in 2020. Our study produces a Full-scale Annual N_2_O dataset in China (FAN2020), providing emergent counting to refine the current national N_2_O inventories.

## Teaser

A four-decade (1980–2020) data of full-scale N_2_O emissions in China was produced. Agricultural N_2_O emissions started to decrease in recent years due to the decline of nitrogen fertilization applications, while, industrial and energetic sources exceeded agriculture and became the fastest-increasing N_2_O sources in China.

## INTRODUCTION

Climate change caused by anthropogenic greenhouse gas (GHG) emissions is one of the global challenges in the 21^st^ century threatening the development of civilizations [1]. Nitrous oxide (N_2_O) is not only one of the most important long-lived GHGs but also the current main stratospheric ozone-depleting substance [2]. The global warming potential of a single molecular N_2_O is about 273 times higher than that of CO_2_ over a 100-year time horizon [3]. The mixing ratio of N_2_O in the atmosphere has increased by 23% from the pre-industry level of 270 ppb to 334 ppb in 2021, and is still increasing at a mean growth rate of 1.0 ppb yr^−1^ [4,5]. Therefore, urgent actions must be conducted to accurately identify N_2_O sources, which further support mitigation strategy making [6].

There are two major components of N_2_O sources: anthropogenic and natural sources. The former includes N_2_O emissions from agriculture, energy, industry, and waste sectors [7]. N_2_O emitted from natural ecosystems is a by-product of nitrogen (N) transformation processes, including nitrification, denitrification, chemodenitrification, and chemical oxidation of hydroxylamine [8,9]. At the global scale, natural sources and anthropogenic sources account for 57% and 43% of the total N_2_O emissions, respectively [6]. Therefore, neither natural nor anthropogenic N_2_O sources should be overlooked in global and regional N_2_O inventories.

Although China is among the emerging economies largely accounting for the growing N_2_O emissions, its full-scale evaluation of national N_2_O inventory has not yet been carried out. The Chinese government has released National Greenhouse Gas Inventories (NGHGIs) five times from 1994 to 2014 (i.e. 1994, 2005, 2010, 2012, and 2014). These inventories quantified anthropogenic N_2_O emissions based on country-level activity data and emission factors (EFs) recommended by the IPCC guidelines on national greenhouse gas inventories (https://unfccc.int/documents). For example, NGHGIs reported 1967 Gg N_2_O yr^−1^ of anthropogenic N_2_O emissions in 2014, which accounts for 17.1% of the global anthropogenic emissions [6,10]. Although NGHGIs provide important insights for understanding China's role in N_2_O emissions, NGHGIs do not include N_2_O emissions from natural sources. As natural sources contribute a substantial fraction of total N_2_O emissions, a comprehensive evaluation involving both anthropogenic and natural sources in China is urgently needed.

In the last four decades, China underwent dramatic changes with respect to population, urbanization, agriculture, industry, and energy consumption, substantially impacting temporal changes in N_2_O emissions. As the most populous nation, China has been challenged to balance the relationship between food production and N fertilizer applications, while the latter acts as the most important anthropogenic N_2_O source. In the last four decades, Chinese N fertilizer applications in agriculture have tripled to meet the food demand of its growing population [11]. The growing rate of N fertilizer applications has started to slow down and a decelerating trend was observed after 2016, owing to the release of the *Zero Growth in Fertilizer Plan* [12]. Meanwhile, fast urbanization has brought a huge increase in waste and wastewater, as well as N_2_O emissions from their treatment [13]. With rapid industrialization, energy consumption has also increased sharply, accelerating both industrial and energetic N_2_O emissions [14]. In addition, atmospheric N deposition, an important driver for N_2_O emission in terrestrial ecosystems, has experienced a dramatic change: N deposition in China has been stable since 2000 [15]. Therefore, a long-term scale study is crucial to reveal the natural and anthropogenic drivers behind national N_2_O emissions.

Here we present a comprehensive synthesis of the Chinese N_2_O budget from 1980 to 2020, including both natural and anthropogenic sources, using IPCC-guided methodology and two state-of-the-art process-based ecosystem models, and these estimates have resulted in the creation of a Full-scale Annual N_2_O dataset (FAN2020). FAN2020 provides additional up-to-date information for improving our evaluation of N_2_O sources and further developing point-targeted policies towards N_2_O mitigation, within the framework of Sustainable Development Goals. In addition, Annex I Parties have been required to report annually their N_2_O emissions to the United Nations Framework Convention on Climate Change (UNFCCC). By the end of 2024, it will be also mandatory for non-Annex I Parties (including China) within the Paris Agreement to report biennially their national GHG inventories with detailed source-determining progress. This study offers a new alternative to estimate the full scale N_2_O emissions in China.

## RESULTS

### Comparisons with NGHGIs and other datasets

**Figure 1.**
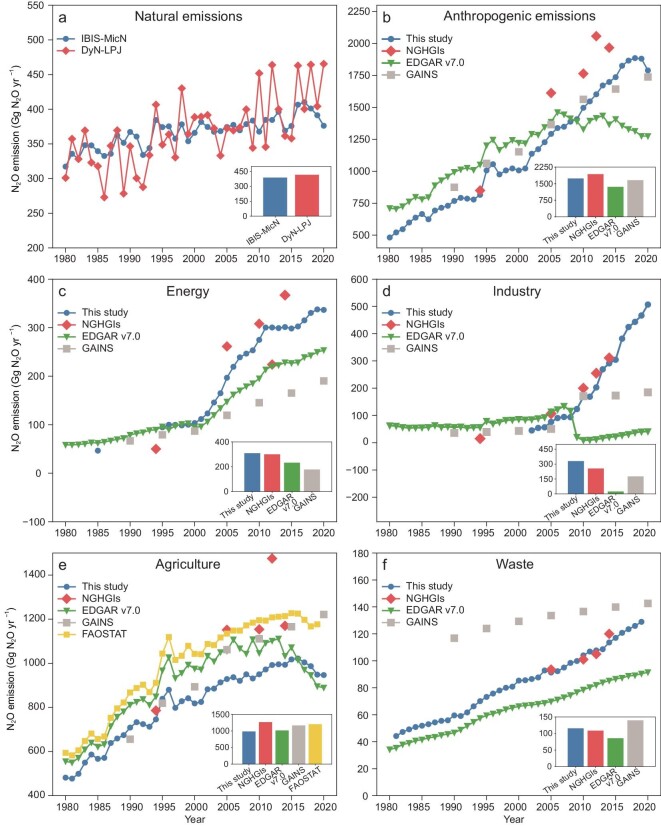
(a–f) Long-term variations of N_2_O emissions from 1980 to 2020 in China for natural and anthropogenic sectors. Bar charts in the right corner represent average values from 2010 to 2020. EDGAR v7.0, the Emissions Database for Global Atmospheric Research; GAINS, the Greenhouse Gas—Air Pollution Interactions and Synergies; FAOSTAT, the Statistics Division of the Food and Agriculture Organization of the United Nations.

Our estimates for the period 1980**–**2020 were generally comparable in magnitude with NGHGIs and other current existing datasets (Fig. 1). For the total anthropogenic N_2_O emissions, the mean value of our estimates (i.e. FAN2020 dataset) in 1994, 2005, 2010, 2012, and 2014 was 1381.5 Gg N_2_O yr^−1^, which was 16.3% lower than that of NGHGIs (Fig. 1b, and Table S1 in the Supplementary Data online). Sector-specifically, our estimates and NGHGIs showed good agreements in energy, industry, and waste sectors (Fig. 1c, d and f), while agricultural N_2_O emissions in our study were 19.6% lower compared with those of NGHGIs (Fig. 1e and Table S1).

Our estimates for the agricultural sector showed good agreements with EDGAR v7.0 and FAOSTAT in respect of temporal dynamics (Fig. 1e). However, the agricultural N_2_O emissions in our study during 1980**–**2020 were 10.0% and 18.1% lower than those of EDGAR v7.0 and FAOSTAT, respectively. In addition, the much lower agricultural N_2_O emissions in our study mainly resulted from the significantly smaller N_2_O emissions from manure management and manure left on pasture (Fig. S1).

Industrial N_2_O emissions of our study agreed well with those of NGHGIs, EDGAR v7.0 and GAINS before 2008, while after 2008 our estimates were still highly in agreement with NGHGIs, but 288.5 Gg N_2_O yr^−1^ and 136.5 Gg N_2_O yr^−1^ higher than EDGAR v7.0 and GAINS, respectively (Fig. 1d). The average N_2_O emissions from waste in this study were 19.0 Gg N_2_O yr^−1^ higher and 39.3 Gg N_2_O yr^−1^ lower compared with those of EDGAR v7.0 and GAINS, respectively (Fig. 1f). During 2000–2020, energetic N_2_O emissions in this study were on average 247.6 Gg N_2_O yr^−1^, which were 61.2 Gg N_2_O yr^−1^ and 106.2 Gg N_2_O yr^−1^ higher than those of EDGAR v7.0 and GAINS, respectively (Fig. 1c).

Natural N_2_O emissions simulated by IBIS-MicN model in this study showed a similar trend but much smaller interannual fluctuation compared with those simulated by DyN-LPJ model (Fig. 1a). However, the mean magnitude of N_2_O emission for natural ecosystems derived from IBIS-MicN model (366.7 Gg N_2_O yr^−1^ from 1980 to 2020) was quite similar to that of DyN-LPJ model (368.1 Gg N_2_O yr^−1^). In addition, the spatial pattern of N_2_O emissions for natural ecosystems simulated by the IBIS-MicN model was similar to that of the DyN-LPJ model (Fig. S2).

### Long-term trends of N_2_O emissions during the last four decades

The total N_2_O emissions of China increased from 889.6 (699.6–1079.6) Gg N_2_O yr^−1^ in 1980 to 2295.0 (1778.6–2811.4) Gg N_2_O yr^−1^ in 2020 (Table 1). From 1980 to 1990, the total N_2_O emissions increased by 38.2%, and agriculture alone contributed 25.5%, which was about 66.7% of the total increase (Table 1). In the 1990s, the N_2_O emission showed the lowest increase rate (i.e. 15.3% decade^−1^) with agriculture acting as the largest contributor. For the decades of 2000**–**2010 and 2010**–**2020, the total N_2_O emissions kept increasing by 31.5% and 23.1%, respectively, while energy and industry exceeded agriculture and became the fastest-increasing N_2_O sources in China. Agricultural N_2_O emissions declined at a pace of 20.5 Gg N_2_O yr^−1^ after 2016, in contrast, natural N_2_O emissions kept increasing steadily at a pace of 1.5 Gg N_2_O yr^−1^ over the last four decades (Fig. 1a).

**Table 1.** Chinese N_2_O emissions in 1980, 1990, 2000, 2010 and 2020.

		N_2_O emissions (Gg N_2_O yr^−1^)				
Sectors	Sources	1980	1990	2000	2010	2020
Natural emissions	Forest	247.9	291.1	296.5	298.9	303.0
	Grassland	69.4	76.2	69.5	68.8	73.3
	**Subtotal**	**317.3**	**367.2**	**366.0**	**367.7**	**376.2**
Energy	Electricity generation	19.2	52.4	61.8	185.9	241.1
	Heat plants	0.5	2.4	3.1	10.2	19.8
	Petroleum refining	0.5	0.1	0.2	0.9	3.4
	Manufacture of solid fuels	1.0	2.2	1.8	5.9	7.2
	Other energy industries	0.1	0.4	0.6	0.3	1.3
	Manufacturing industries and construction	15.1	24.7	23.9	45.5	36.6
	Transport	4.1	4.2	5.3	17.1	20.0
	Residential	4.3	5.7	4.3	5.6	4.5
	Agriculture/forestry/fishing/fish farms	0.9	1.1	1.2	1.4	1.7
	Non-specified	0.2	1.0	0.9	2.0	0.8
	Fugitive emissions from fuels	0.6	0.3	0.3	0.1	0.0
	**Subtotal**	**46.5** **^a^**	**94.6** **^a^**	**103.4**	**274.8**	**336.6**
Industry	Nitric acid production			10.6	38.3	38.7
	Adipic acid production			34.5	129.8	468.0
	**Subtotal**			**45.1** **^a^**	**168.1**	**506.7**
Agriculture	Fertilizer application in cropland	197.7	305.4	356.8	479.5	413.1
	Nitrogen mineralization	82.1	122.6	124.7	124.3	170.0
	Manure left on pasture and manure management	79.7	99.7	119.3	91.4	91.7
	Manure application in cropland	39.0	49.7	71.7	78.5	64.9
	Crop residue	30.8	49.1	49.6	59.5	70.6
	Nitrogen deposition	31.7	46.1	49.8	56.8	81.5
	Fertilizer and manure application in pasture	7.7	14.3	20.8	30.4	25.9
	Nitrogen leaching/runoff	10.7	17.9	21.6	25.1	23.5
	Biomass burning	2.3	3.6	3.8	4.5	5.3
	**Subtotal**	**481.5**	**708.4**	**818.1**	**950.1**	**946.6**
Waste	Biological treatment of solid waste and waste incineration			0.1	2.0	10.1
	Wastewater treatment and discharge	44.2	59.5	85.3	102.1	118.8
	**Subtotal**	**44.2** **^a^**	**59.5**	**85.5**	**104.0**	**128.9** **^a^**
**Anthropogenic total**		**572.2**	**862.6**	**1052.1**	**1497.1**	**1918.8**
**Total**		**889.6**	**1229.8**	**1418.0**	**1864.8**	**2295.0**

a Due to a lack of N_2_O emissions from the energy sector in 1980 and 1990, this table uses values in 1985 and 1995. Similarly, the values of N_2_O emission from the industrial sector in 2002 and from the waste sector in 1981 and 2019 are used to replace the values in 2000, 1980 and 2020, respectively.

### Sectoral N_2_O source profile in China

**Figure 2.**
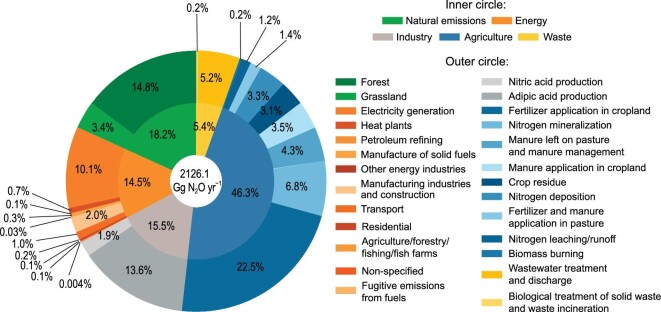
Share of each source in Chinese N_2_O emissions (2010–2020).

Total N_2_O emissions from all sectors in China were 2126.1 Gg N_2_O yr^−1^ for 2010**–**2020, with 18.2% (387.6 Gg N_2_O yr^−1^) from natural emissions, and the remaining 81.8% (1738.5 Gg N_2_O yr^−1^) from anthropogenic emissions (Fig. 2). Forests were responsible for more than 80% (314.3 Gg N_2_O yr^−1^) of the total natural N_2_O sources. Agricultural N_2_O emissions represented 46.3% of the total emissions (Fig. 2) and 56.6% of the anthropogenic emissions, which were very close to those (59.5%) reported in NGHGIs in 2014 [10]. Fertilizer applications in agriculture accounted for almost half of the total agricultural N_2_O sources, followed by N mineralization, manure left on pasture and manure management, manure application, and N deposition (Fig. 2). In contrast, approximately 5 Gg N_2_O yr^−1^ of biomass burning turned out to be the smallest agricultural N_2_O source.

Industry accounted for the third largest (15.5%) N_2_O source in China. Adipic acid and nitric acid production accounted for 87.8% and 12.2% of the total industrial N_2_O emissions, respectively (Fig. 2). Energy and waste contributed 14.5% and 5.4% to the total emissions, respectively. About 70% of energetic N_2_O sources came from electricity generation, followed by manufacturing and construction. In comparison, N_2_O emissions from petroleum refining, other energy industries, and fugitive emissions from fuels were almost neglectable. Biological treatment of solid waste and waste incineration, as well as wastewater treatment and discharge, were the two main N_2_O sub-sources within the waste. N_2_O emitted from wastewater treatment and discharge mounted up to 110.9 Gg N_2_O yr^−1^, which was responsible for more than 95% of the total N_2_O emissions from waste (Fig. 2).

### Spatial N_2_O source profile in China

**Figure 3.**
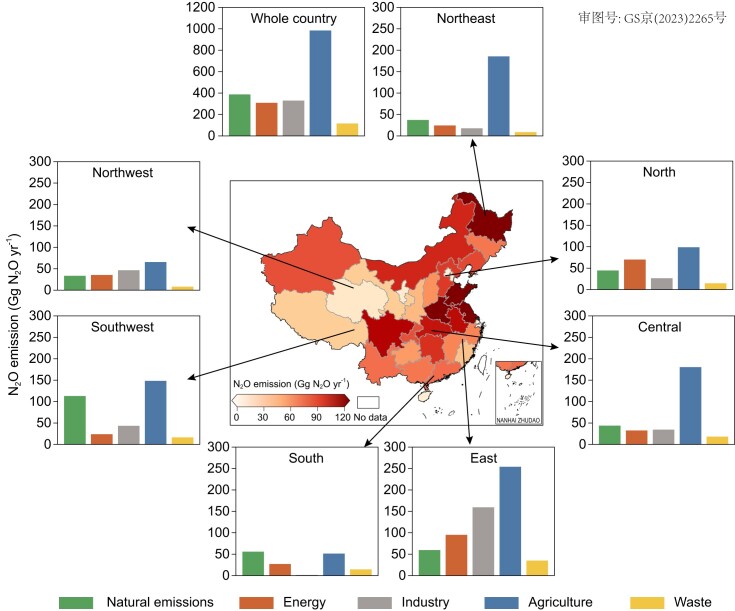
Regional N_2_O emissions in China (2010–2020). The mainland of China is partitioned into seven regions: Northwest, Southwest, South, East, Central, North and Northeast. Each subplot shows the emissions from five sectors. The center map shows the spatial pattern of the decadal mean (2010–2020) of total N_2_O emissions from all sectors. Administrative divisions for seven regions in the mainland of China are shown in Fig. S11. Data from Hong Kong, Macau, and Taiwan of China are not available in this study.

N_2_O emissions differed largely across provinces. Agricultural-developed regions generally accounted for higher N_2_O emissions (Fig. 3). The East of China, contributing 28.4% (603.2 Gg N_2_O yr^−1^) of the national N_2_O emissions in China during the last decade, turned out to be the largest regional N_2_O source. The Southwest became the second-largest regional N_2_O source with 345.2 Gg N_2_O yr^−1^, while the smallest N_2_O emissions of 150.5 Gg N_2_O yr^−1^ occurred in the South of China.

Agriculture accounted for the largest N_2_O source in all regions except for the South. The sum of agricultural N_2_O emissions from the Northeast, East, Central, and Southwest exceeded 75% of the national agricultural emissions (Fig. 3). By contrast, natural N_2_O emissions were large in the Southwest with over 100 Gg N_2_O yr^−1^, but much smaller in the other regions with about 50 Gg N_2_O yr^−1^. The largest N_2_O emission from waste, energy, and industry occurred in the East, where there is the highest population density in China. Large natural N_2_O emissions in the Southwest were found in Yunnan (Fig. S3a). Shandong, Jiangsu, and Anhui were responsible for the high agricultural emissions in the East (Fig. S3e). The hot spots of N_2_O emission from industry in the East were mainly observed in Shandong and Jiangsu (Fig. S3d).

**Figure 4.**
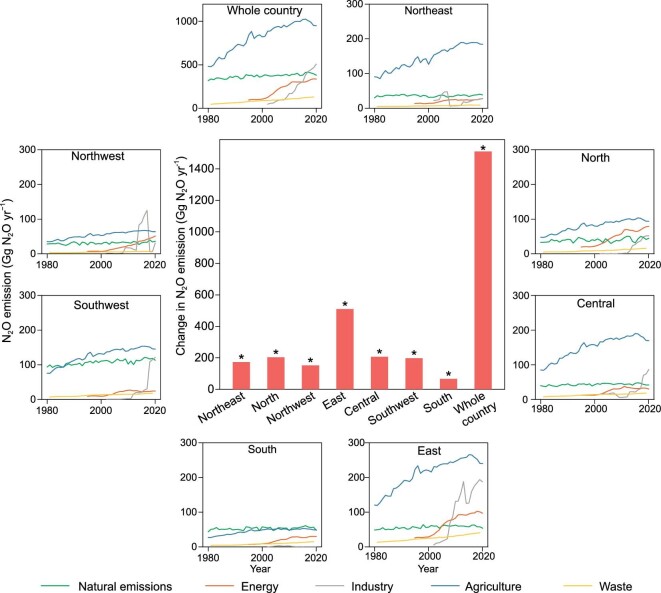
National and Regional changes of each N_2_O source from 1980 to 2020. The bar chart in the center shows the accumulated changes of regional and national N_2_O emissions in China from 1980 to 2020. The accumulated changes were calculated from the linear regressed annual trend (Gg N_2_O yr^−1^) multiplied by 41 years. The Mann-Kendall test was used to test a significantly increasing trend in N_2_O emissions for each region and the whole country during the period of 1980**–**2020. All regions show a significant increasing trend in N_2_O emissions over the study period. *P < 0.05. Administrative divisions for seven regions in the mainland of China are shown in Fig. S11.

The East did not only represent the largest N_2_O emissions but also the fastest increasing trend of 12.4 Gg N_2_O yr^−1^ among all the seven Chinese regions (Fig. 4). The Central, North, and Southwest showed increasing trends of 5.0, 5.0, and 4.8 Gg N_2_O yr^−1^, respectively. Changes in natural N_2_O emissions in all seven regions were very steady, while N_2_O emissions from energy increased rapidly in the East, North, and Northwest after 2000. No significant increase in N_2_O emissions from industry was observed in the South, but it increased by four times in the East from the 2000s to the 2010s (Fig. 4).

## DISCUSSION

### Reliability of FAN2020

Compared with other inventories, FAN2020 reports much lower agricultural N_2_O emissions (Fig. 1e), largely due to the adaption of EFs and parameters in manure left on pasture and manure management, and indirect N_2_O emissions from N leaching/runoff. On one hand, the adaption of EF_3_, N excretion rates of livestock, fraction of manure in management systems brought some of inconsistency. EF_3_ of manure left on pasture was adapted from 0.02 kg N_2_O-N kg^−1^ N in IPCC 2006 to 0.004 kg N_2_O-N kg^−1^ N in IPCC 2019 for cows, swine, and poultry, and from 0.01 kg N_2_O-N kg^−1^ N to 0.003 kg N_2_O-N kg^−1^ N for sheep and other livestock (Table S2) [7,16]. In the meantime, N excretion rates of goat and sheep decreased to 0.34 kg N (1000 kg animal mass)^−1^ day^−1^ from 1.37 kg N (1000 kg animal mass)^−1^ day^−1^ and to 0.32 kg N (1000 kg animal mass)^−1^ day^−1^ from 1.17 kg N (1000 kg animal mass)^−1^ day^−1^, respectively (Table S3) [7,16]. In addition, the fraction of manure in management systems was quantified in detail in IPCC 2019, while largely omitted in IPCC 2006 (Table S3) [7,16]. The adaption of these parameters decreased N_2_O emission estimates by 13–30.7 Gg N_2_O yr^−1^ for manure management and by 70.6–123.5 Gg N_2_O yr^−1^ for manure left on pasture (Fig. S4), accounting for part of the inconsistency of our FAN2020 with other datasets including EDGAR v7.0, GAINS, and FAOSTAT (Fig. S1). On the other hand, the remaining difference is mainly due to the adaption of EF_5_ and the fraction of N loss by leaching and runoff to N applications. EF_5_ used in this study was derived from Zhou *et al.* [14], which was 0.0065 kg N_2_O-N (kg N leaching and runoff)^−1^ (i.e. the sum of the EF values from different leaching and runoff systems). In contrast, EF_5_ were 0.011 kg N_2_O-N (kg N leaching and runoff)^−1^ in IPCC 2019 and 0.0075 kg N_2_O-N (kg N leaching and runoff)^−1^ in IPCC 2006 [7,16]. Moreover, this study estimated the fraction of N loss by leaching and runoff to N applications ranging from 1.21–8.27% for paddy rice and 0.61–8.74% for upland crops. On the contrary, most of the other datasets used the default fractions of 24% and 30% by IPCC 2019 and IPCC 2006, respectively [7,16].

Moreover, FAN2020 adapted EF_1_ (see Table S4, emission factors for agricultural synthetic fertilizer and manure application, N deposition, and N mineralization) into six climate regions according to Zhou *et al.* [14] to improve the accuracy instead of simply using IPCC Tier 1. In IPCC 2019, EF_1_ was defined as 0.01 kg N_2_O-N kg^−1^ N for upland and 0.004 kg N_2_O-N kg^−1^ N for paddy without considering regional climate effect [7]. According to Zhou *et al.* [14], the EF_1_ show obvious regional differences along the whole country: EF_1_ for upland was relatively high in the East (0.0157 kg N_2_O-N kg^−1^ N) and Northeast (0.0149 kg N_2_O-N kg^−1^ N), but lower in other regions (0.0065–0.0093 kg N_2_O-N kg^−1^ N), and EF_1_ for paddy ranged from 0.0052 kg N_2_O-N kg^−1^ N to 0.0161 kg N_2_O-N kg^−1^ N in China, which was relatively higher than that of 0.004 kg N_2_O-N kg^−1^ N in IPCC 2019 [7]. Optimization of EF_1_ increased the regional resolution of agricultural N_2_O emissions (Fig. S5).

Incomparable N_2_O emission abatement strategies and activity data are reasons for the differences of industrial N_2_O emissions among inventories. EDGAR v7.0 assumed that adipic acid plants in China implemented abatement technologies under the Clean Development Mechanism (CDM) to reduce N_2_O emissions [17], while GAINS followed the suggestion by Schneider *et al.* [18] that abatement was not implemented for the new adipic acid plants [19]. N_2_O abatement was also considered in NGHGIs [20,21], but the activity data they collected from the enterprise survey suggested that adipic acid production expanded largely in the meantime [22]. Based on the CDM reports during 2008**–**2012 (https://cdm.unfccc.int/), this study assumed that only Henan Shenma Nylon Chemical Co., Ltd and PetroChina Company Limited Liaoyang Petrochemical Co. installed N_2_O abatement techniques. High uncertainty was introduced owing to the discrepancy in understanding of the operation of abatement devices.

Our estimate of average N_2_O emissions from natural sources is comparable with a previous estimate of 368.1 ± 49.5 Gg N_2_O yr^−1^ by the DyN-LPJ model (Fig. 1a) [23]. Apart from the autotrophic nitrification and denitrifier denitrification considered in the DyN-LPJ model, the IBIS-MicN model included additional N_2_O-producing processes of heterotrophic nitrification and nitrifier denitrification [24], therefore, the IBIS-MicN model performed with a much smaller inter-annual variability. Both natural N_2_O emissions from forests and grasslands in this study are within the range of previous reports using the direct extrapolation method [25–28], linear models [29], random forest regression model [30], or process-based models [31,32].

### Implications for understanding N_2_O budget in China

Anthropogenic and natural activities dominating N_2_O emissions have experienced substantial changes in the last four decades, such as N fertilizer application [33], N deposition [15], energy use, and industry development [34], which largely changed N_2_O emissions. Globally, natural sources account for about 57% of the total N_2_O emissions [6], while, our results showed that natural sources only account for 18.2% of the total N_2_O emissions in China, and anthropogenic activities account for the remaining 81.8% (Fig. 2). Larger N_2_O emissions from the anthropogenic sources than those from natural sources in China can be explained by the excessive N fertilizer applications, large production of chemical industry, and extensive fuel consumption. This implies the large mitigation potential of anthropogenic N_2_O emissions in China.

To develop sustainable agriculture, the Chinese government began to make efforts to enhance the use efficiency of N fertilizer. In 2015, the Chinese Ministry of Agriculture issued the *Action Plan for Zero Growth in Fertilizer Use by 2020*. Afterwards, huge efforts have been made to reduce fertilizer applications while optimizing agricultural management to increase crop yields including: (1) adjustment of fertilizer structure through changing the proportion of N, phosphorus, potassium, and trace elements; (2) optimization of fertilization management with right fertilizer type, right amount, right placement strategy, and right application time; (3) recycling of manure and cropland residues to replace chemical fertilizers [12]. As a result, Chinese agricultural N applications decreased by 13.6% from 35.4 Tg N yr^−1^ in 2016 to 30.6 Tg N yr^−1^ in 2020 (Fig. S6a), and agricultural N_2_O emissions declined by 7.3% in China in the meantime (Fig. 1e), which is comparable with a previous report [11]. Even so, agriculture still stands for the largest anthropogenic N_2_O emissions, implying existing practices need to be strengthened and further abatement actions are needed, such as boosting N use efficiency by using enhanced-efficiency fertilizers and organic amendments, optimizing livestock manure management through the shift from solid manure systems to liquid manure systems.

Abatement technologies like thermal or catalytic reduction can remove 80%**–**98% of N_2_O emitted from adipic acid and nitric acid production [16,21,35]. N_2_O abatement equipment has been installed for adipic acid plants since the 1990 s in Europe, resulting in an 98% reduction in industrial N_2_O emissions between 1990 and 2017 [36]. Reductions in industry N_2_O emissions have also been observed globally over the past three decades due to the installation of abatement devices in adipic acid plants [6,17,36]. However, only two plants with N_2_O abatement in China have been reported in CDM projects so far [18]. As the first commitment period under the *Kyoto Protocol* only covered the years 2008**–**2012, the commitment of N_2_O abatement from these two plants has not been reported in CDM after 2012; thus, it is unclear whether these plants have continued to reduce N_2_O emissions due to the high cost of abatement [37,38]. Additionally, there has been a rising demand for adipic acid production in China, leading to high N_2_O emissions in the last decade (Fig. 1d). This implies that cost-effective abatement is urgently required in China. The end treatment technologies like the thermal decomposition method should be considered to reduce N_2_O emissions from industry. Since there would be extra costs for chemical plants to install and operate the abatement equipment, government subsidies can make plants more motivated to abate N_2_O emissions.

During the combustion of fossil fuels, part of petrogenic N is thermally catalyzed into N_2_O, and its EFs are controlled by the composition of fossil fuels [7,16]. Rapid increases in coal usage are the most important cause for the large increase rate of energetic N_2_O emission in the 2000s (Fig. S7 and Table 1). In the 2010s, the Chinese government implemented a number of policies and measures to optimize energy structure by reducing coal usage [39,40]. Afterward, the growth rate of energetic N_2_O emission slowed down in the 2010s (Table 1). To achieve carbon neutrality, further adjustment of energy structure by cutting down coal usage and increasing the use of clean energy can not only reduce N_2_O emissions from the energy sector, but also significantly reduce CO_2_ emissions.

Waste N_2_O emission highly depends on population growth and the process of urbanization [16], which introduce large amounts of waste containing N compounds into the environment, which lead to the production of N_2_O compounds during waste treatment [41]. The growth rate of the Chinese population during the last 10 years (7.5 million person yr^−1^) was only about half of that in the 1980s (15.5 million person yr^−1^, Fig. S6b), and the increasing rate of waste N_2_O emissions slowed down (Fig. 1f). Even so, with the growing need for wastewater treatment in the future, more advanced technologies of wastewater treatment (e.g. denitrification biofilters) are needed to be developed to abate N_2_O emissions from waste.

In natural ecosystems, N_2_O is primarily produced as a by-product during the biotic and abiotic processes of N transformation [9]. The N_2_O emission rates from natural ecosystems change rapidly at temporal and spatial scales affected by N deposition, atmospheric CO_2_ concentration, and climate [23,42,43]. Through simulation experiments described in Table S5, we found that atmospheric N deposition was the dominant driving force, increasing natural soil N_2_O emissions by 38.0 Gg N_2_O yr^−1^, during the period 1980–2020 (Fig. S8). Previous studies based on a meta‐analysis [44], a conceptual model [29], and an ensemble of terrestrial biosphere model showed that atmospheric N deposition enhances natural soil N_2_O emissions [45], which was consistent with our results. Apart from the effect of atmospheric N deposition, climate change leaded to an increase of 1.6 Gg N_2_O yr^−1^ for soil N_2_O emissions (Fig. S8), which can be explained by soil-atmosphere feedback mechanisms [46,47]. Rising soil temperature can promote the activities of denitrifiers and nitrifiers, further enhancing the microbial N_2_O production rate [48]. However, atmospheric CO_2_ concentrations are observed to reduce soil N_2_O emissions by 12.3 Gg N_2_O yr^−1^ through CO_2_ fertilization effects [45,49]. Rising atmospheric CO_2_ concentration brings CO_2_ fertilization effect stimulating vegetation N uptake, which reduces inorganic N concentration in soil. Less inorganic N concentration in soil will suppress soil N_2_O emissions. Similarly, previous studies through process-based model simulations also suggested that atmospheric CO_2_ fertilization suppressed natural soil N_2_O emissions [23,45,50,51]. Although atmospheric CO_2_ concentration reduced soil N_2_O emissions, the contribution of this negative effect only accounts for one-third of the increase in N deposition-induced N_2_O emissions. With the exacerbation of anthropogenic N deposition [52], a further acceleration of N_2_O emissions is expected in the future.

The Chinese government has mandated that each province should create its own road maps for reducing GHG emissions [53], making it crucial to evaluate the N_2_O emissions as a first step in effectively mitigating them [54]. Our results showed large differences in N_2_O sources among provinces (Fig. S3), offering an important basis for local government to develop province-specific N_2_O-mitigating strategies.

### Reducing uncertainties

Although the FAN2020 dataset provides insights for understanding the N_2_O budget in China, there exists still non-negligible uncertainties (Figs S9 and S10). Uncertainties of activity data and EFs account for most of the uncertainties of anthropogenic N_2_O sources. On one hand, most of the activity data in this study were collected from the provincial statistical system resulting in low uncertainties (Fig. S10a). However, activity data of several categories are neither included in the national nor provincial statistical system, they can only be collected from literature or other incomplete database (Table S6). For example, there is no statistical dataset for adipic acid and nitric acid production, which makes it hard to compare the reliability of FAN2020, NGHGIs, and other inventories. With the growing demand to precisely evaluate GHG budgets, the activity data related to GHG emissions should be included in the national and provincial statistical systems.

On the other hand, EFs and other parameters related to N transformation are just as important as activity data in accurately quantifying anthropogenic N_2_O sources [55]. According to our uncertainty assessment, the variation of EFs and N-transforming parameters brought more than 20% uncertainties of the total N_2_O emission uncertainties (Fig. S10b). For example, the IPCC guidelines reported a large uncertainty of EF (400%) for wastewater treatment and discharge owing to insufficient field experiment data [16]. In addition, the IPCC default EFs and N-transforming parameters are inadequate for capturing the variations across various climate regions, agricultural management methods, combustion technologies, as well as mitigation practices. Specific methodologies, country/regional, technology-specific EFs, and a range of simple to complex process-based models have been developed to address this problem. However, this study used the global default EFs to estimate N_2_O emissions from energy, industry, and waste sectors because country-specific EFs are currently unavailable. Therefore, more accurate EFs in China for these three sectors are needed to reduce uncertainties in emission estimation. Likewise, the agricultural sector has an urgent requirement for country-specific EFs to better reflect the climatic and soil variability and production systems [54,56]. This study used country-specific EFs generated by Zhou *et al.* [14] to estimate N_2_O emissions in the agriculture sector. However, the EFs only represent spatial variations. Numerous studies have shown strong impacts of climate change on N_2_O emissions [57,58]. Thus, further improvements of EFs are still needed to ensure they can indicate the temporal variations with climate change and management. This study has used two process-based ecosystem models (i.e. IBIS-MicN and DyN-LPJ) to evaluate N_2_O emission of the natural sector, and future studies will make efforts to examine the reliability of the process-based ecosystem model for the agricultural sector.

In addition to the natural land mentioned above, aquatic ecosystems, including reservoirs, streams, and rivers, are also important N_2_O sources [59,60]. The total N_2_O emissions from global rivers and streams account for 10%–15% of the total anthropogenic N_2_O emissions [61], and global riverine N_2_O emissions went through a growth of 91.5% from 235.7 Gg N_2_O yr^−1^ in 1961 to 455.7 Gg N_2_O yr^−1^ in 2016 [62]. N inputs into aquatic ecosystems include N leaching, N deposition, wastewater discharge, local N mineralization, etc., and aquatic N_2_O emissions are consistent with the geographic distribution of N use and population [60,63]. Unfortunately, aquatic N_2_O emissions in China are still missing in all the current inventories and, therefore need to be incorporated into future inventories of China.

Our results based on extensive databases and models fill the current gaps in natural and anthropogenic N_2_O sources at national and regional levels. To reduce national N_2_O emissions, efforts should be taken to promote the efficiency of N fertilizer use in agriculture, optimize manure management strategy, implement industrial N_2_O abating strategies, and advance waste treatment technologies. We also highlight that special care should be put into the choice of activity data and EFs when N_2_O inventories are created under the guidance of IPCC Tier 1 and Tier 2. The implementation of N_2_O abatement for adipic acid and nitric acid plants influences their corresponding EFs by multiple times, but this information is nearly unavailable at both national and regional levels. Future research is needed to expand the current database of activity data and develop more targeted EFs based on local conditions and N_2_O-producing processes.

## DATA AND METHODS

### Estimation of anthropogenic N_2_O sources

Four anthropogenic N_2_O sectors (agriculture, energy, industry, and waste) were calculated following the IPCC 2019 methodology [7]. The N_2_O emissions (E) of each sector were estimated at the province level according to the following equation:


(1) \begin{eqnarray*}
E( y) = \mathop \sum \limits_i \mathop \sum \limits_j \left[ {A{D}_{i,j}\left( y \right) \times E{F}_i} \right],
\end{eqnarray*}

where $A{D}_{i,j}( y )$ indicates the activity data of source *i* in province *j* for the year of *y*; $E{F}_i$ is the EF for source *i*. Detailed calculations of each sector are described in Sections S1.1–1.4 in the Supplementary Data online.

### Estimation of natural N_2_O sources

Two process-based N_2_O emission models, the IBIS-MicN and DyN-LPJ models, were applied to quantify N_2_O fluxes across natural ecosystems including forests and grasslands. In IBIS-MicN, N_2_O emissions were simulated from four microbial N_2_O-producing processes (i.e. autotrophic nitrification, heterotrophic nitrification, nitrifier denitrification, and denitrifier denitrification) based on the dynamic activities of nitrifiers and denitrifiers, as well as local environmental conditions simulated by IBIS [24]. While DyN-LPJ simulated N_2_O emissions from autotrophic nitrification and heterotrophic denitrification based on the fully coupled carbon and N dynamics [23]. Several model experiments were conducted to quantify the factorial contributions of atmospheric CO_2_ concentration (CO_2_), atmospheric N deposition (N_dep_), and climate change (CLIM) on natural N_2_O emissions from 1980 to 2020 (Table S5). Details of the two models were described in Section S1.5.

### Uncertainty analysis

The uncertainty of anthropogenic N_2_O emissions was assessed through the uncertainties of activity data, EFs, and other related parameters for the calculation (Section S1.6).

The uncertainty of natural N_2_O emissions simulated by the IBIS-MicN model was assessed through the Markov chain Monte Carlo (MCMC) method (Section S1.6).

### Full-scale annual N_2_O dataset for China

This study integrated the above two estimates of N_2_O emission from both anthropogenic and natural N_2_O sources and generated a Full-scale Annual N_2_O dataset (FAN). FAN includes N_2_O emission in five sectors: agriculture, energy, industry, waste, and natural land (Table S7). Most sectors cover the temporal variations of N_2_O emissions from 1980 to 2020, and hence the version was named FAN v2020. For anthropogenic sources, FAN follows the categories of each sector in the IPCC-guided methodology and includes 24 categories for all anthropogenic sources, which is estimated at the provincial level. Natural sources include N_2_O emission from forests and grasslands simulated at a spatial resolution of 25 km×25 km.

### Comparison with other existing inventories

This study also included several existing N_2_O emission datasets for comparison with our estimates: NGHGIs, EDGAR v7.0, GAINS, and FAOSTAT, which were developed following the IPCC Tier 1 approach (Table S8). A detailed description of all four inventories was in Section S2.

## Supplementary Material

nwad285_Supplemental_File

## Data Availability

The dataset constructed by this study is publicly available at https://doi.org/10.57760/sciencedb.07961.
