# Supplementary material for: Four decades of full-scale nitrous oxide emission inventory in China
Source: Natl Sci Rev. 2023 Nov 6;11(3):nwad285. doi: 10.1093/nsr/nwad285 (PMC10939392; doi:10.1093/nsr/nwad285)
Supplement: nwad285_Supplemental_File [file nwad285_supplemental_file.docx]

**Supplementary Information**

Section S1: Methods for calculating N_2_O emissions from each source

**S1.1 N_2_O emissions from agriculture**

S1.1.1 Application of synthetic fertilizer and manure

Annual N_2_O emissions due to the application of synthetic fertilizer (*N_2_O_fer_*, kg N_2_O yr^-1^) and manure (*N_2_O_man_*, kg N_2_O yr^-1^) in cropland ecosystem for each province were calculated following Eq. S1 and S2, respectively:

${N_{2}O}_{fer}=N_{fer}\times{EF}_{1}\times\frac{44}{28}$ (Eq. S1)

${N_{2}O}_{man}=N_{man}\times{EF}_{1}\times\frac{44}{28}$ (Eq. S2)

where $N_{fer}$ and $N_{man}$ (kg N yr^-1^) are annual amounts of synthetic fertilizer and manure applied to cropland and pasture, which were derived from the HaNi dataset with a spatial resolution of 5-arcmin ^[1]^. EF_1_ is a region-specific emission factor (kg N_2_O-N kg^-1^ N) proposed by Zhou et al ^[2]^ (Table S4). 44/28 is the conversion coefficient of N_2_O-N emissions to N_2_O emissions. Since the HaNi dataset was generated based on the Land-Use Harmonization (LUH2) dataset (<https://luh.umd.edu/data.shtml>), the LUH2 dataset was used to identify the distribution of cropland and pasture. Provincial N_2_O emissions were calculated by summing all pixels in each province, and provincial distributions of paddy and upland were calculated according to the *China Statistical Yearbook*. Since the HaNi dataset covered the N deposition and application dataset from 1980 to 2019, the values of 2020 were estimated according to the linear regression from 2017 to 2019.

S1.1.2 N mineralization

Annual N_2_O emissions due to N mineralization (*N_2_O_min-cr_*, kg N_2_O yr^-1^) in cropland were calculated following Eq. S3 to Eq. S4:

${N_{2}O}_{min-cr}=N_{min-cr}\times{EF}_{1}\times\frac{44}{28}$ (Eq. S3)

$N_{min-cr}=N_{min}\times P_{cr}$ (Eq. S4)

where *N_min-cr_* (kg N yr^-1^) is the annual N mineralization of cropland in each province; *N_min_* (kg N yr^-1^) is the average rate of soil N mineralization over the entire province simulated by Integrated BIosphere Simulator (IBIS) model; *P_cr_* (%) stands for the proportion of cropland to all land use types.

S1.1.3 Crop residue

Annual N_2_O emissions due to residue (*N_2_O_cr-re_*, kg N_2_O yr^-1^) in cropland were calculated following Eq. S5 to Eq. S8:

${N_{2}O}_{cr-re}=CRN\times{EF}_{1}\times\frac{44}{28}$ (Eq. S5)

$CRN=\sum_{i}^{T} ({AGN}_{(i)}+{BGN}_{(i)})$ (Eq. S6)

${AGN}_{(i)}={Yield}_{(i)}\times{DRY}_{(i)}\times R_{AG\left( i \right)}\times{P_{Re}\times N}_{AG\left( i \right)}$ (Eq. S7)

${BGN}_{(i)}={Yield}_{\left( i \right)}\times{DRY}_{\left( i \right)}\times(1+R_{AG\left( i \right)})\times{RS}_{\left( i \right)}\times N_{BG\left( i \right)}$ (Eq. S8)

where *CRN* (kg N yr^-1^) is N input from residues of all crops; *AGN_(i)_* (kg N yr^-1^) and *BGN_(i)_* (kg N yr^-1^) are N input from above-ground and below-ground residues of the *i*th crop, respectively; *Yield_(i)_* (kg yr^-1^) stands for the annual statistical production of the *i*th crop, derived from *China Statistical Yearbook*; *DRY_(i)_* stands for dry matter fraction of the harvest grain of the *i*th crop; *R_AG(i)_* stands for the dry matter ratio of above-ground residue to yield; *N_AG(i)_* (kg N kg ^-1^) and *N_BG(i)_* (kg N kg ^-1^) represent the N content of above-ground and below-ground residues, respectively; *RS_(i)_* (kg kg ^-1^) represents the ratio of below-ground biomass to above-ground biomass. Values of the above parameters were derived from IPCC (Table S9) ^[3]^. *P_Re_* is the fraction of above-ground residues remaining in cropland, derived from the literature (Table S10). This study included 10 main crops (i.e., T=10), including rice, wheat, maize soybean, potato, other grain, peanut, rapeseed, sugar beet, and sugarcane. Their production accounts for 98.50% of total production in China according to the statistical data from the *China Statistical Yearbook* in 1980**–**2020.

S1.1.4 Biomass burning

Annual N_2_O emission due to biomass burning (*N_2_O_cr-burn_*, kg N_2_O yr^-1^) in cropland was calculated following Eq. S9 and Eq. S10:

${N_{2}O}_{cr-burn}=\sum_{i}^{T} M_{burn(i)}\times{EF}_{burn}$ (Eq. S9)

$M_{burn(i)}={Yield}_{\left( i \right)}\times{DRY}_{\left( i \right)}\times R_{AG\left( i \right)}\times P_{burn}$ (Eq. S10)

where *M_burn(i)_* (kg yr^-1^) is dry matter burnt annually of the *i*th crop; *EF_burn_* (kg N_2_O kg^-1^ dry matter burnt ) represents the emission factor of residue burning, which was derived from IPCC (Table S4) ^[3]^; *P_burn_* indicates the fraction of above-ground residues burnt in each province (Table S10).

S1.1.5 Livestock excretion and manure management

Annual N_2_O emissions due to livestock excretion and manure management (*N_2_O_MS_*, kg N_2_O yr^-1^) were calculated following Eq. S11 to Eq. S13:

${N_{2}O}_{MS}=\sum_{i}^{T} \sum_{j}^{S} {MS}_{(i,j)}\times{EF}_{3(i,j)}\times\frac{44}{28}$ (Eq. S11)

${MS}_{(i,j)}=N_{(i)}\times N_{exc(i)}\times P_{(i,j)}$ (Eq. S12)

$N_{exc(T)}=N_{rate(T)}\times\frac{T_{AM\left( T \right)}}{1000}\times365$ (Eq. S13)

where *MS_(i, j)_* (kg N yr^-1^) is the annual N excretion of the *i*th livestock in pasture with the *j*th manage system; Livestock species considered in this study consist of dairy cattle, other cattle, goat, sheep, horse, mule, asses, camel, swine, and poultry; *N_(i)_* (animal) is the number of the *i*th livestock in a given year, which is derived from *China Statistical Yearbook*. It should be noted that *N_(i)_* for poultry was calculated with the annual production divided by 12 months and multiplied by the growth time of 2 months. *N_exc (i)_* (kg N animal^-1^ yr^-1^) is annual N excretion per head of the *i*th livestock; *P_(i, j)_* is fraction of manure in the *j*th management system accounting for total manure of the *i*th livestock; *EF_3(i, j)_* (kg N_2_O-N kg^-1^ N) stands for emission factor for direct manure excretion or manure management (Table S4); *N_rate(T)_* (kg N (1000kg animal mass)^-1^ day^-1^) stands for N excretion rate of livestock *T*; *T_AM(T)_* (kg animal^-1^) stands for mass of livestock *T*. Values of above parameters were from IPCC ^[3]^ (Table S3).

S1.1.6 Indirect emissions from N deposition

Annual N_2_O emissions due to N deposition (*N_2_O_dep-cr_*, kg N_2_O yr^-1^) in cropland were calculated following Eq. S14 to Eq. S15:

${N_{2}O}_{dep-cr}=N_{dep-cr}\times{EF}_{1}\times\frac{44}{28}$ (Eq. S14)

$N_{dep-cr}=N_{dep-agr}\times P_{cr-arg}$ (Eq. S15)

where *N_dep-cr_* (kg N yr^-1^) indicates annual N deposition on cropland; *N_dep-agr_* (kg N yr^-1^) is annual N deposition for the pixel derived from the HaNi dataset^[1]^, and *P_cr-agr_* (%) stands for the proportion of cropland to both pasture and cropland which was derived from the LUH2 dataset ^[4]^.

S1.1.7 Indirect emissions from N leaching and runoff

Annual indirect N_2_O emissions from N leaching and runoff (*N_2_O_LR_*, kg N_2_O yr^-1^) were calculated following Eq. S16 to Eq. S17:

${N_{2}O}_{LR}=\sum_{i}^{S} N_{input}\times F_{leach}\times{EF}_{5(i)}\times\frac{44}{28}$ (Eq. S16)

$N_{input}=N_{fer}+N_{man}+N_{min-cr}+CRN+MS$ (Eq. S17)

where *N_input_* (kg N yr^-1^) is annual soil N input; *F_leach_* (%) is the fraction of N loss by leaching and runoff to N applications on managed soils (Table S11); *EF_5(i)_* (kg N (kg N leaching and runoff)^-1^) represents emission factor for N_2_O emissions from the *i*th N leaching and runoff system (Table S4).

**S1.2 N_2_O emissions from energy sector**

S1.2.1 Energy consumption

Annual N_2_O emissions from combustions of fuels (*N_2_O_CF_*, kg N_2_O yr^-1^) were calculated following Eq. S18:

${N_{2}O}_{CF}=\sum_{i}^{S} \sum_{j}^{T} {EC}_{(i,j)}\times{EF}_{fuel(j)}\times{CF}_{(j)}$ (Eq. S18)

where *EC_(i, j)_* (Gg yr^-1^) is energy consumption from the *i*th source for the *j*th fuel type from energy balance statistics from *China Energy Statistical Yearbook*. We considered 10 sources including electricity generation, heat plants, petroleum refining, manufacture of solid fuels, other energy industries, manufacturing industries and construction, transport, residential, agriculture/forestry/fishing/fish farms, and hon-specified. *EF_fuel(j)_* (kg N_2_O TJ^-1^) represent the emission factors for energy consumption and were listed in Table S4; *CF_(j)_* (TJ Gg^-1^) is the conversion factor for the *j*th fuel type, listed in Table S12. N_2_O emissions from coal electricity generation were computed using the Tier 3 methods based on the IPCC Guidelines ^[5]^, because the process of electricity generation from coal using the configuration of circulating bed and bubbling bed leads to extremely high N_2_O emissions.

S1.2.2 Fugitive emissions from fuels

Annual fugitive N_2_O emissions from fuel (*N_2_O_FE_,* kg N_2_O yr^-1^) were calculated following Eq. S19:

${N_{2}O}_{FE}=\sum_{i}^{T} F_{(i)}\times{EF}_{fug(i)}$ (Eq. S19)

where *F_(i)_* (Gg yr^-1^) is the energy activity (e.g., waste gas flaring) that causes fugitive emissions for the *i*th fuel type obtained from *China Energy Statistical Yearbook*; *EF_fug(i)_* (kg N_2_O Gg^-1^) represents fugitive N_2_O emission factor for the *i*th fuel type (Table S4).

**S1.3 N_2_O emissions from industry**

Annual N_2_O emissions from the chemical industry (*N_2_O_PC_*, kg N_2_O yr^-1^) were calculated following Eq. S20:

${N_{2}O}_{PC}=\sum_{i}^{T} \sum_{j}^{P} P_{(i, j)}\times{EF}_{ci(i)}-{N_{2}O}_{AE(j)}$ (Eq. S20)

where *P_(i, j)_* (t yr^-1^) is production of the *i*th chemical industry from the *j*th chemical plant from websites for plants, chemical productions including nitric acid and adipic acid production; *EF_ci(i)_* (kg N_2_O t^-1^) represents emission factor for direct N_2_O emission from production of the *i*th chemical industry; *N_2_O_AE(j)_* (kg N_2_O yr^-1^) is annual N_2_O abatement emission for the *j*th chemical plant from Clean Development Mechanism (CDM; <https://cdm.unfccc.int/>).

**S1.4 N_2_O emissions from waste**

S1.4.1 Biological treatment of solid waste

Annual N_2_O emissions from biological treatment of solid waste (*N_2_O_solid_*, kg N_2_O yr^-1^) were calculated following Eq. S21:

${N_{2}O}_{solid}=\sum_{i}^{T} {SW}_{(i)}\times{EF}_{solid(i)}$ (Eq. S21)

where *SW_(i)_* (kg yr^-1^) is solid waste composted for the *i*th waste from *China Urban-Rural Construction Statistical Yearbook*; *EF_solid(i)_* (kg N_2_O kg^-1^) represents emission factor for biological treatment of the *i*th solid waste (Table S4).

S1.4.2 Waste incineration

Annual N_2_O emissions from waste incineration (*N_2_O_inc_*, kg N_2_O yr^-1^) were calculated following Eq. S22:

${N_{2}O}_{inc}=WI\times{EF}_{inc}$ (Eq. S22)

among which, *WI* (Gg yr^-1^) is waste incineration from *China Urban-Rural Construction Statistical Yearbook*; *EF_inc_* (kg N_2_O Gg^-1^ wet waste) represents the emission factor for waste incineration and was listed in Table S4.

S1.4.3 Wastewater treatment and discharge

Annual N_2_O emissions from wastewater treatment and discharge (*N_2_O_wt_*, kg N_2_O yr^-1^) were calculated following Eq. S23 and Eq. S24:

${N_{2}O}_{wt}=N_{wt}\times{EF}_{wt}\times\frac{44}{28}$ (Eq. S23)

$N_{wt}=(P\times Protein\times F_{NPR}\times F_{NonCon}\times F_{IndCom})-N_{Sludge}$ (Eq. S24)

where *N_wt_* (kg N yr^-1^) is annual N contained in wastewater; *P* (capita) is human population from *China Statistics Yearbook*; *Protein* (kg capita ^-1^ yr^−1^) is annual per capita protein consumption from Food and Agriculture Organization of the United Nations (FAO; <http://faostat.fao.org/>); *F_NPR_* (kg N kg^-1^ protein) is fraction of N in protein set as default (= 0.16 kg N kg^-1^ protein); *F_NonCon_* is the factor for non-consumed protein added into the wastewater set as 1.4; *F_IndCom_* is the factor for industrial and commercial co-discharged protein into the sewer system set as 1.25; *N_Sludge_* is N removed with sludge set as 0. Values of the above parameters were from IPCC ^[3]^. *EF_wt_* (kg N_2_O-N kg N^-1^) represents the emission factor for wastewater treatment and discharge.

**S1.5 N_2_O emission from natural sources**

The dynamic activities of nitrifiers and denitrifiers, as well as the ammonium and nitrate contents in the soil, were simulated in the IBIS-MicN model. Further, N_2_O emissions were calculated from four microbial N_2_O-producing processes, i.e., autotrophic nitrification, heterotrophic nitrification, nitrifier denitrification, and denitrifier denitrification. A previous study showed a good performance of the IBIS-MicN model in reproducing the spatial and temporal variations of N_2_O emissions of forest and grassland ecosystems ^[6]^. IBIS-MicN model has been validated by observation data from 30 global forest and grassland sites with *R*^2^ being 0.85. Here we further validated the IBIS-MicN model using 23 sets of N_2_O field measurements from Xu et al. 2018 ^[7]^ (Table S13). We found that the annual values of modeled and observed N_2_O emissions were highly correlated with *R*^2^ being 0.85 at 23 forest and grassland validation sites in China (Fig. S12), which illustrated a good performance in simulating natural N_2_O emissions in China. DyN-LPJ was developed to estimate N_2_O emissions within terrestrial ecosystems by integrating with the Lund-Potsdam-Jena (LPJ) dynamic global vegetation model ^[8]^. Apart from the fully coupled vegetation and carbon dynamics based on LPJ, DyN includes several important processes related to N_2_O emission: nitrification, denitrification, plant N uptake, allocation and turnover, plant and soil N mineralization, biological N_2_ fixation, NH_3_ volatilization, N leaching, as well as N_2_, N_2_O, and NO production and emission. N_2_O emission from natural vegetation in China simulated by DyN-LPJ has been validated by 28 sets of N_2_O field measurements and showed a good performance in simulating N_2_O emission from natural vegetation in China ^[7]^.

A gridded daily meteorological dataset produced using the thin-plate spline method was used to drive the IBIS-MicN model in this study ^[9]^, including air temperature, precipitation, cloud fraction, wind speed, air pressure, and relative humidity. Meteorological observations at 735 meteorology stations from the National Climate Center of China Meteorological Administration were used to interpolate the gridded climate dataset with a spatial resolution of 25×25 km. Monthly N deposition was required from the IGAC/SPARC Chemistry‐Climate Model Initiative (<https://esgf-node.llnl.gov/search/input4mips/>). Soil pH was provided by the global soil property datasets from a global soil dataset for earth system modeling ^[10]^. The distribution of natural ecosystems was derived from China's Land-Use/Cover Datasets ^[11]^. All input data were resampled to the same spatial resolution as the meteorological dataset using the nearest interpolation method. Simulated results by the IBIS-MicN model were further compared to that of another process-based DyN-LPJ N_2_O emission model.

We conducted several model experiments to quantify the factorial contributions of atmospheric CO_2_ concentration (CO_2_), atmospheric N deposition (N_dep_), and climate change (CLIM) on natural N_2_O emissions from 1980 to 2020 (Table S5). The control experiment S0 maintained all drivers at their 1980 level. The other simulation experiments (S1-S4) each had one additional changing driver compared to the preceding experiment. Contributions from individual drivers were identified by comparing results from different simulation experiments. For example, the contribution of CO_2_ was estimated by subtracting the simulated result of the S0 simulation from those in the S1 simulation.

**S1.6 Uncertainty analysis**

The uncertainties of the anthropogenic sources were assessed following the IPCC guidelines. The uncertainty of the N_2_O emission from a specific source, *u_emi_* (%), was calculated following Eq. S25 ^[3]^:

$u_{emi}=\sqrt{{{(u}_{AD}}^{2}+{u_{EF}}^{2})}$ (Eq. S25)

where *u_AD_* (%) is the uncertainty of activity data, and *u_EF_* (%) is the uncertainty of EFs and related parameters. The total uncertainties of all sources *u_total_* _(%)_ were calculated following Eq S26:

$u_{total}=\frac{\sqrt{\sum_{i}^{S} {(u_{emi(i)}\times E_{(i)})}^{2}}}{\sum_{i}^{S} E_{(i)}}$ (Eq. S26)

where *E_(i)_* (Gg N_2_O yr^-1^) is the N_2_O emission for the *i*th N_2_O source. The uncertainty for each type of activity data was listed in Table S14, and uncertain ranges of EFs and other N_2_O-emittion related parameters were listed in Table S4 and Table S15.

Uncertainties in natural N_2_O emissions were evaluated through a parameter uncertainty analysis ^[12]^. Based on our previous optimization of model parameters through the Markov chain Monte Carlo (MCMC) method, we have calculated averages and standard deviations of the major parameters that control N_2_O flux. We first assumed that the distribution of each parameter followed a normal distribution. Second, we randomly selected an ensemble of multiple sets of the major parameters in affecting N_2_O flux simulated by the IBIS-MicN model. Finally, this ensemble of the major parameters was used to simulate N_2_O flux, which can reflect the uncertainty of model parameters. More information on averages and standard deviations of the major parameters can be found in the reference (Ma et al. 2022 ^[6]^).

Section S2 Datasets used for comparison

**S2.1 NGHGIs**

NGHGIs report the national GHG emission estimates. China submitted its Initial, Second, and Third National Communications on Climate Change in 2004, 2012, and 2018 respectively, and provided 1994, 2005, and 2010 National GHG Inventories. In addition, the First and Second Biennial Update Report on Climate Change of the People’s Republic of China were submitted in 2016 and 2018 respectively, with the 2012 and 2014 National GHG Inventories. The NGHGIs anthropogenic N_2_O emissions include emissions from four key sectors in China: energy, industry, agriculture, and waste. The National GHG Inventories reported that N_2_O emissions of land use, land-use change, and forestry (LULUCF) were very small, and almost close to zero. The estimation methods in the respective sectors follow the approach provided by IPCC 2006 Guidelines for National Greenhouse Gas Inventories ^[10]^. These guidelines provided detailed descriptions of methods for estimating emissions. The emissions were computed using the Tier 1 or Tier 2 methods in the IPCC Guidelines. Activity data are mainly from the *China Statistical Yearbook* and other relevant statistics provided by the National Bureau of Statistics. Some of EFs were determined by statistical surveys, and others were defined using default values from the IPCC Guidelines.

**S2.2 EDGAR v7.0**

EDGAR is a global emission inventory including greenhouse gases and air pollutants built by the Joint Research Centre of the European Commission (<https://edgar.jrc.ec.europa.eu/index.php>). The version of the EDGAR product is updated annually and reports data starting in 1970 until the prior year. EDGAR dataset covers all anthropogenic N_2_O emission sectors at the annual level. Anthropogenic N_2_O emissions were calculated using activity data from statistical data (e.g., FAO, International Energy Agency, United States Geological Survey, etc.), and EFs from IPCC guidelines. In addition, monthly emissions were provided by the sector with a spatial resolution of 0.1° × 0.1°. The latest datasets (EDGAR v7.0) span from 1970 to 2021 (<https://edgar.jrc.ec.europa.eu/emissions_data_and_maps>).

**S2.3 GAINS**

GAINS model estimated emissions of 6 GHGs at the province level based on activity data from the international energy database and industrial statistics, and information supplied by provinces themselves. Source sectors and EFs in GAINS were defined by the IPCC Tier 1 or Tier 2 methodology. Almost all anthropogenic N_2_O emission sectors (energy, industry, agriculture, and waste) were included in GAINS except for emissions from biomass burning and land use/land use change. GAINS provided province-specific emission estimates in China spanning a time period from 1990 to 2050 in 5-year intervals (<https://gains.iiasa.ac.at/gains/docu.EAN/index.menu?open=none>).

**S2.4 FAOSTAT**

FAOSTAT: The Statistics Division of the Food and Agriculture Organization of the United Nations, provides the total N_2_O emission from agriculture ([https://www.fao.org/faostat/en/#data/GT](https://www.fao.org/faostat/en/%23data/GT)). Emissions were calculated following the IPCC Guidelines at the Tier 1 level. Activity data were obtained from countries reporting to FAO about livestock, crops, and fertilizers used in agriculture. The main N_2_O emission sources were reported in FAOSTAT including manure management, synthetic fertilizers, manure applied to the soils, manure left in pasture, crop residues, cultivation of organic soils, burning-savanna, and burning crop residues. Emission estimates are available by country and for the period 1961**–**2019 with annual updates. Specifically, emission data from “Cultivation of organic soils” and “Burning-savanna” started in 1990.

**S2.5 Comparison of data and methods used in this study with other four inventories**

Differences among N_2_O-emitting sectors, study period, and spatial resolution of FAN2020 and other inventories were listed in Table S8.

Section S3: Supplement figures and tables


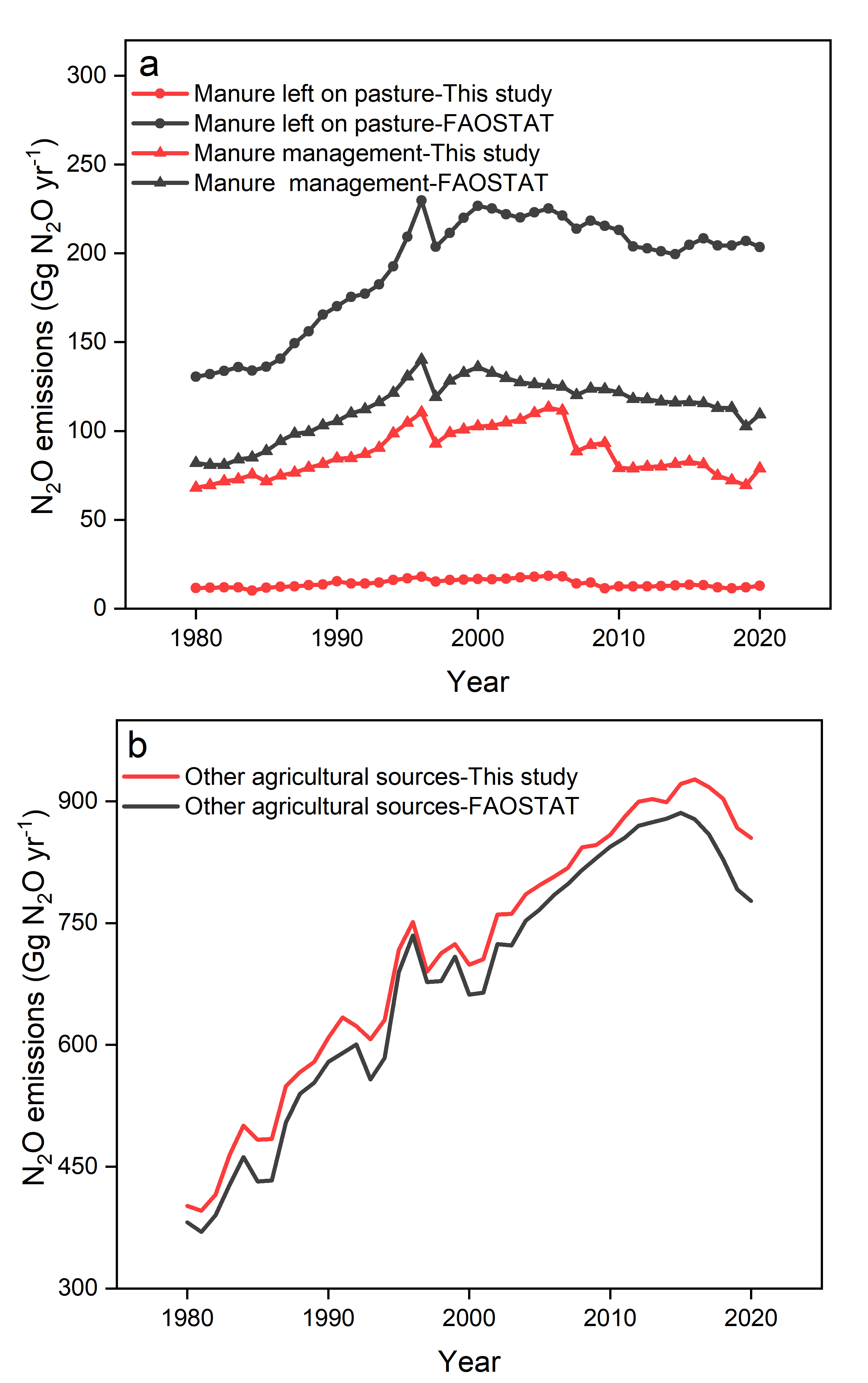


**Figure S1.** Comparison of N_2_O emissions from manure left on pasture and manure management (a), and other agricultural sources (b) in this study and FAOSTAT.


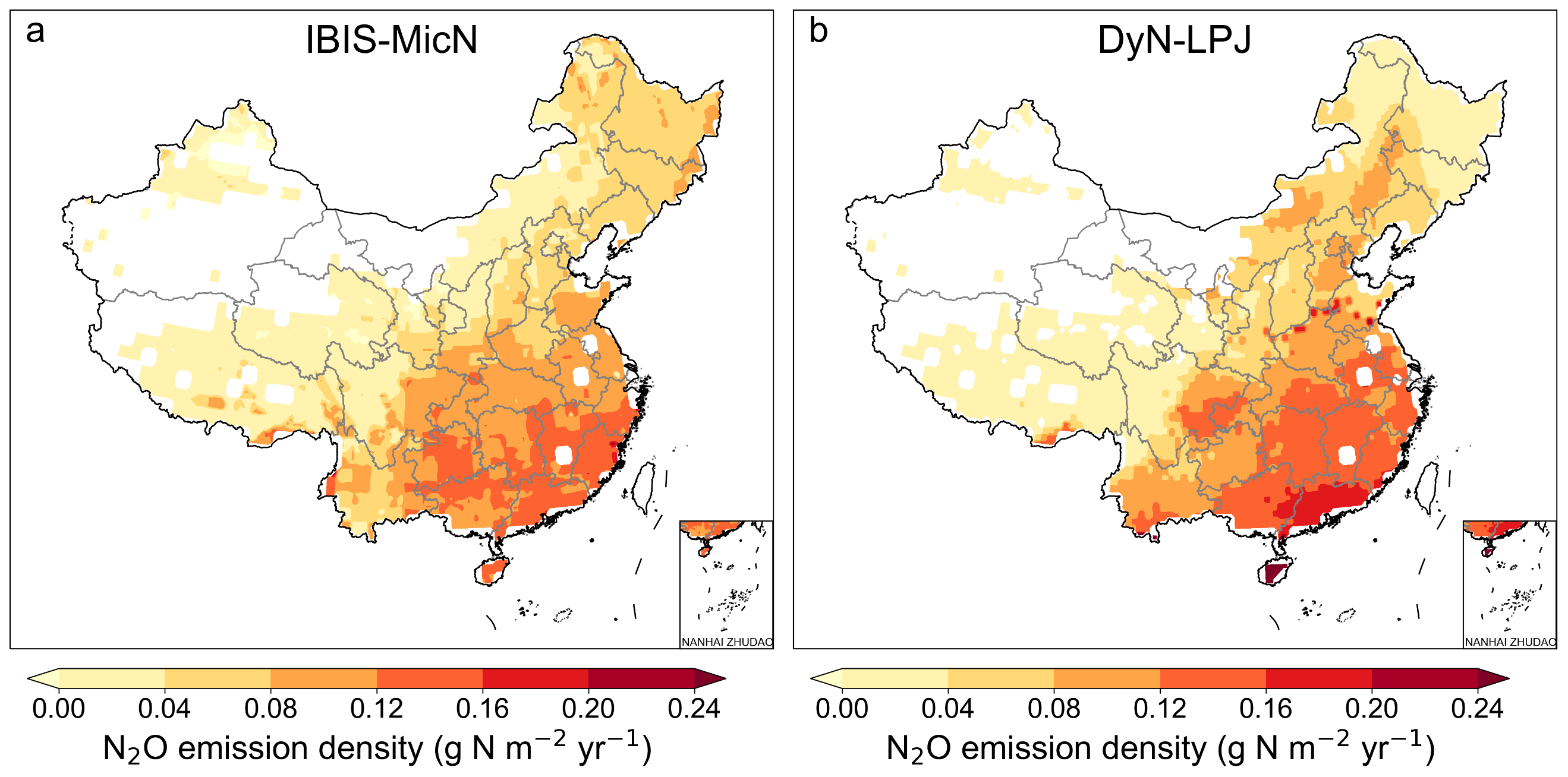


**Figure S2.** The spatial pattern of decadal mean (2010**–**2020) of natural N_2_O emission density simulated by IBIS-MicN (a) and DyN-LPJ (b). Data from the Hong Kong, Macao, and Taiwan are not available in this study.


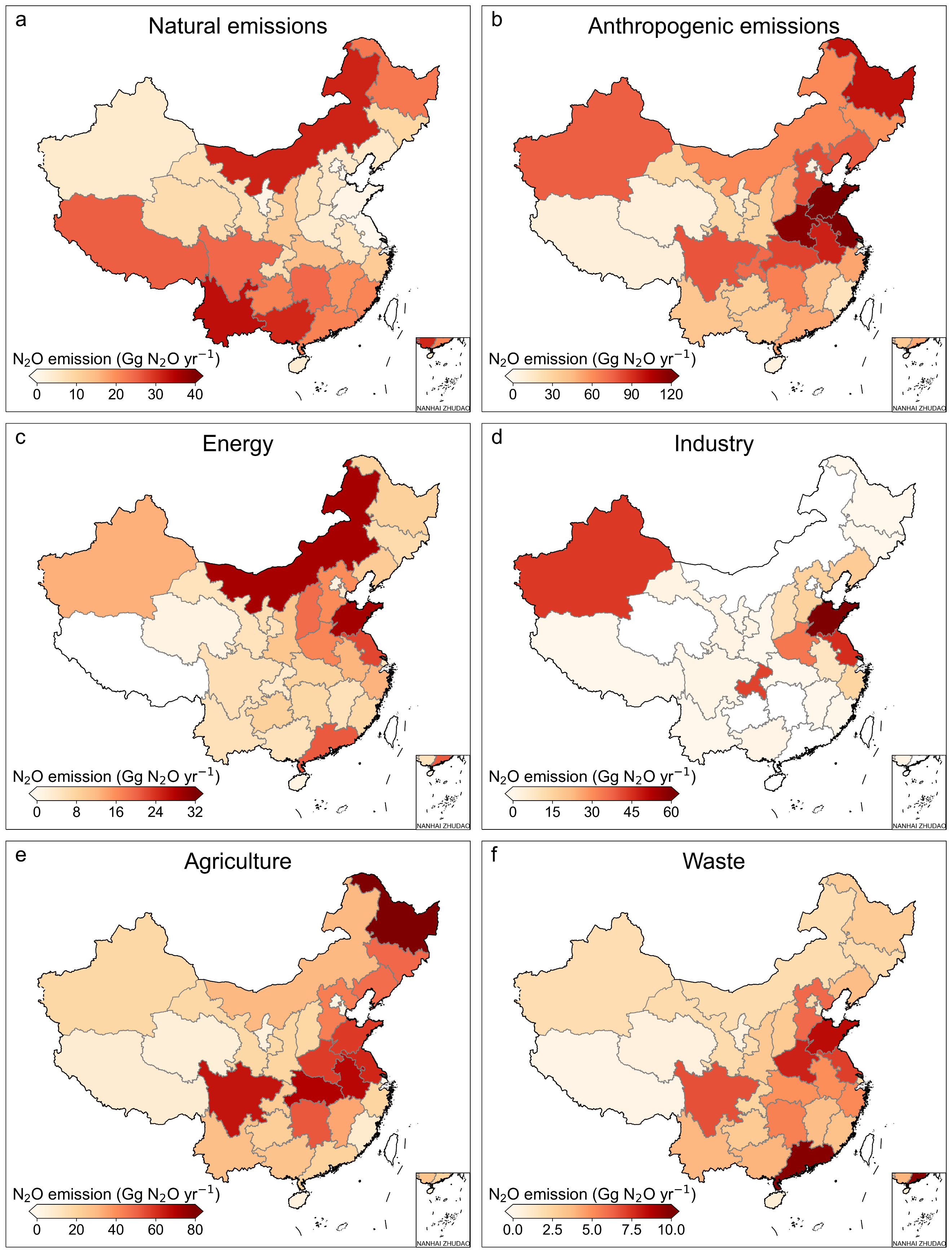


**Figure S3.** The spatial pattern of decadal mean (2010**–**2020) of N_2_O emissions from each sector. Data from the Hong Kong, Macao, and Taiwan are not available in this study.


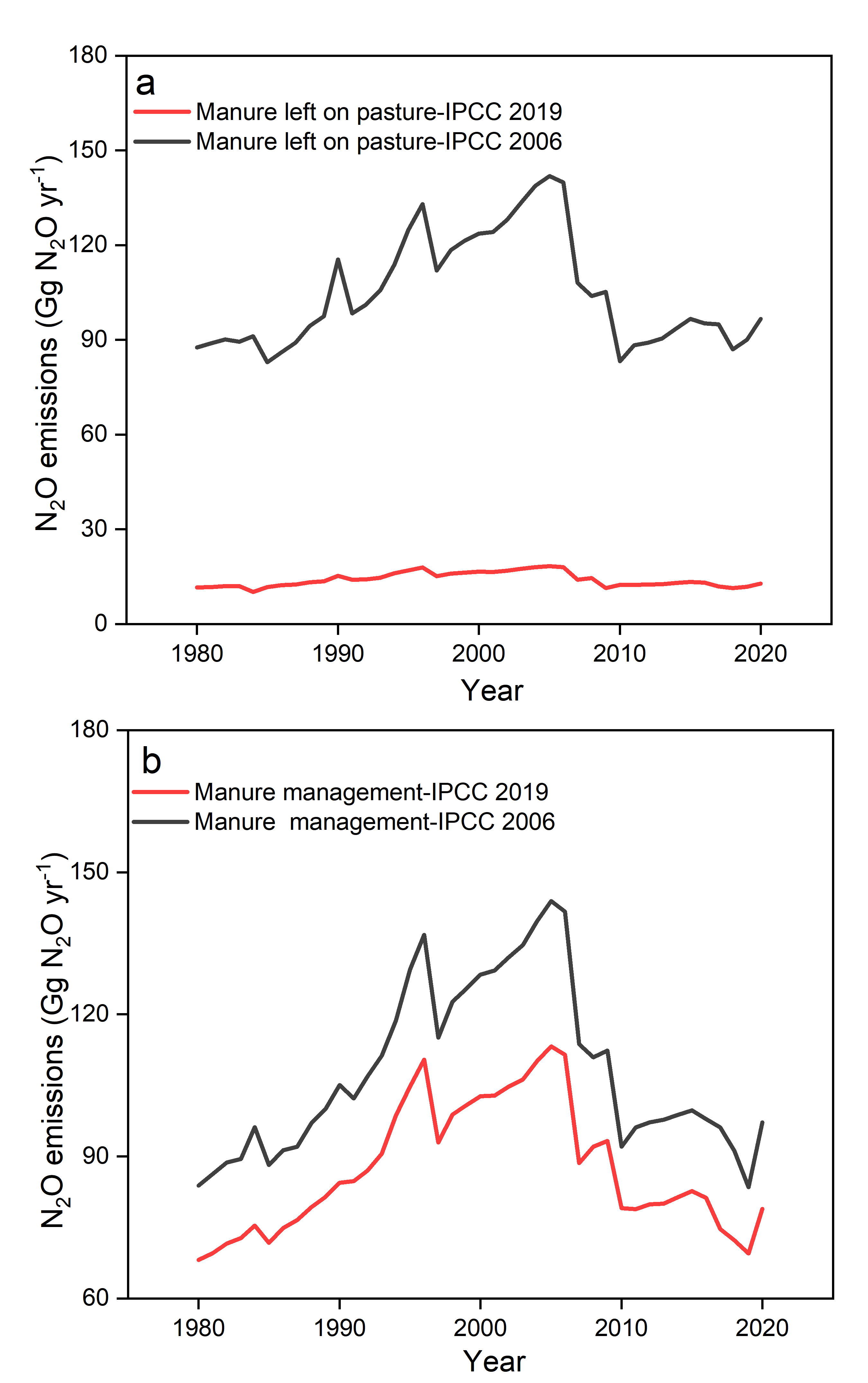


**Figure S4.** Differences of N_2_O emissions from manure left on pasture (a) and manure management (b) calculated according to parameters of IPCC 2019 and IPCC 2006.


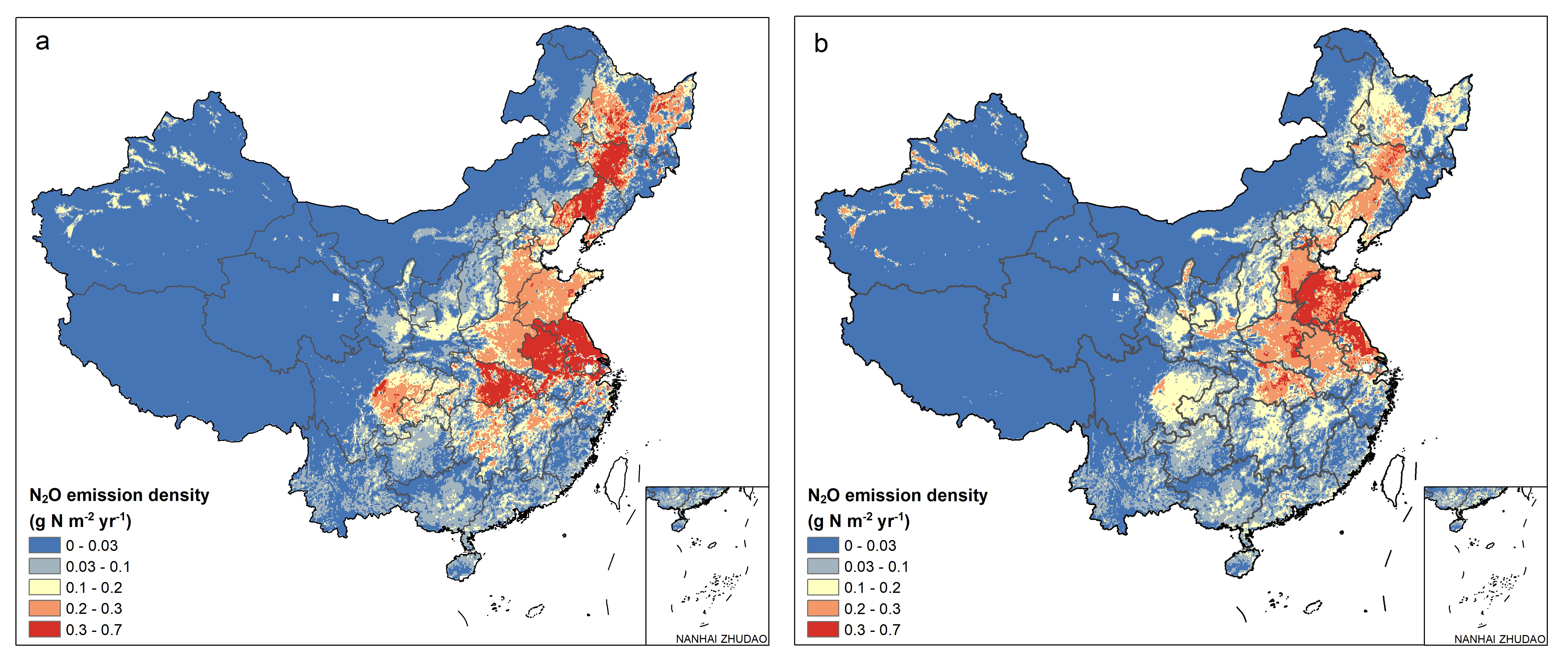


**Figure S5.** The spatial pattern of N_2_O emissions in 2015 from fertilizer and manure application on cropland, calculated based on recommended EFs by in this study (a) and IPCC 2019 (b). Data from the Hong Kong, Macao, and Taiwan are not available in this study.


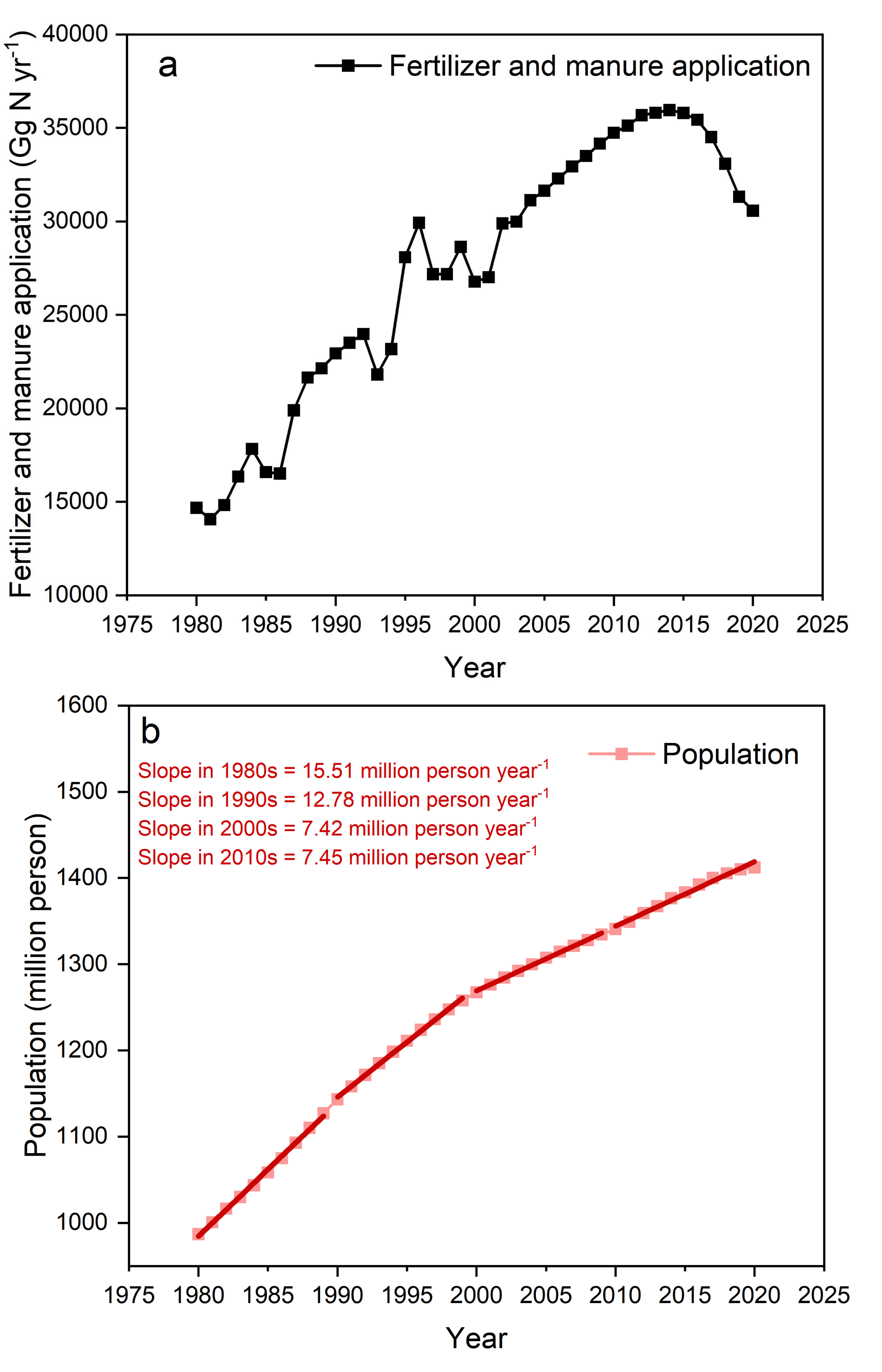


**Figure S6.** Fertilizer and manure application (a) and growth of population (b) from 1980 to 2020. Data from FAOSTAT and *China Statistical Yearbook*, respectively.





**Figure S7.** Dynamics of energy consumption in coal, petroleum and natural gas during 1980**–**2020.

**

**

**Figure S8.** Contributions of factors (climate [CLIM], N deposition [N_dep_], and CO_2_ concentration [CO_2_]) to natural soil N_2_O emissions from 1980 to 2020.


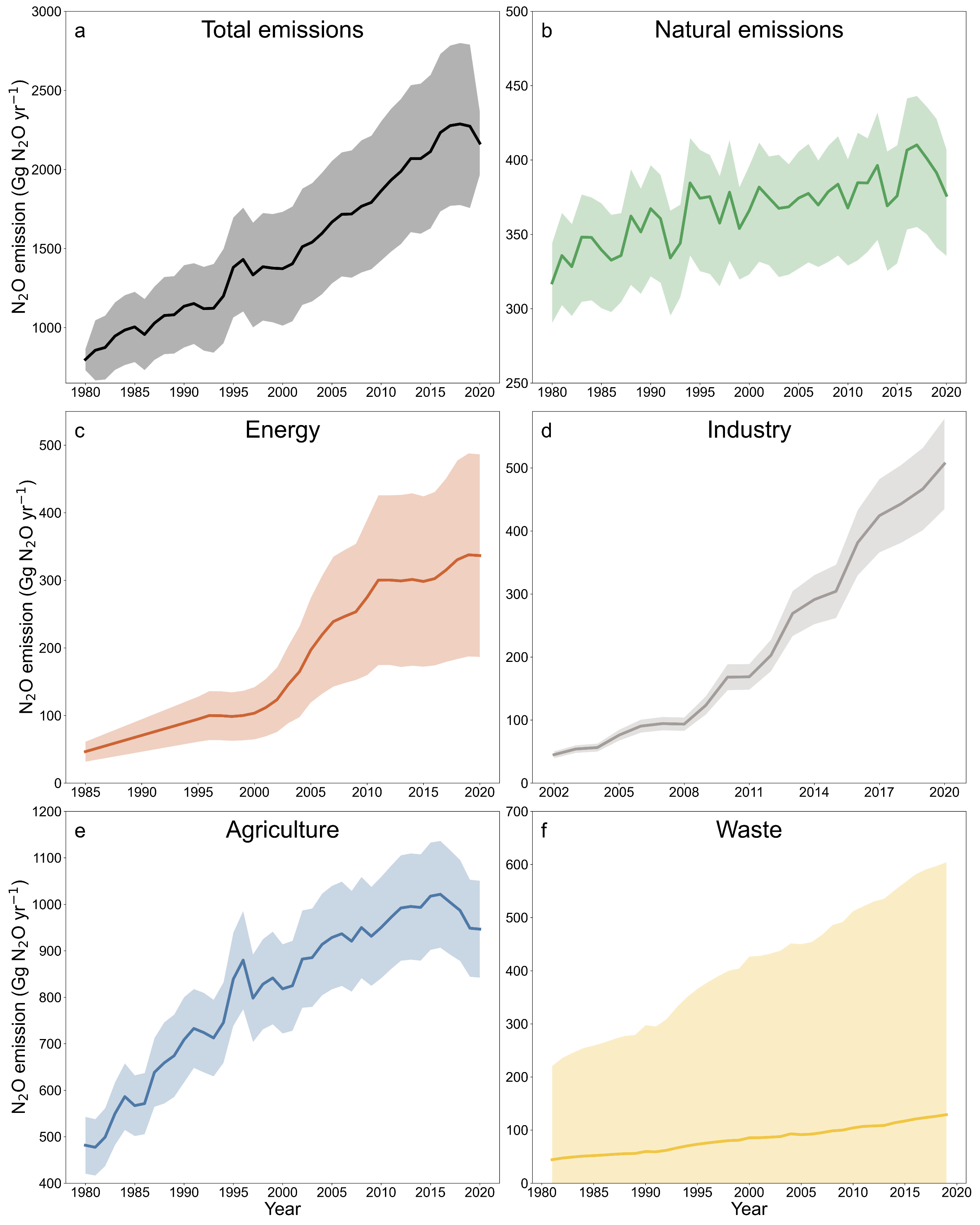


**Figure S9.** Long-term variations of anthropogenic and natural N_2_O emissions with their uncertainties (the shaded area) in China.





**Figure S10.** Uncertainties of anthropogenic N_2_O emissions (2010–2020) resulted from activity data (a), EFs and related parameters (b), and both of them (c). The source code corresponding to the source name can be found in Table S7.


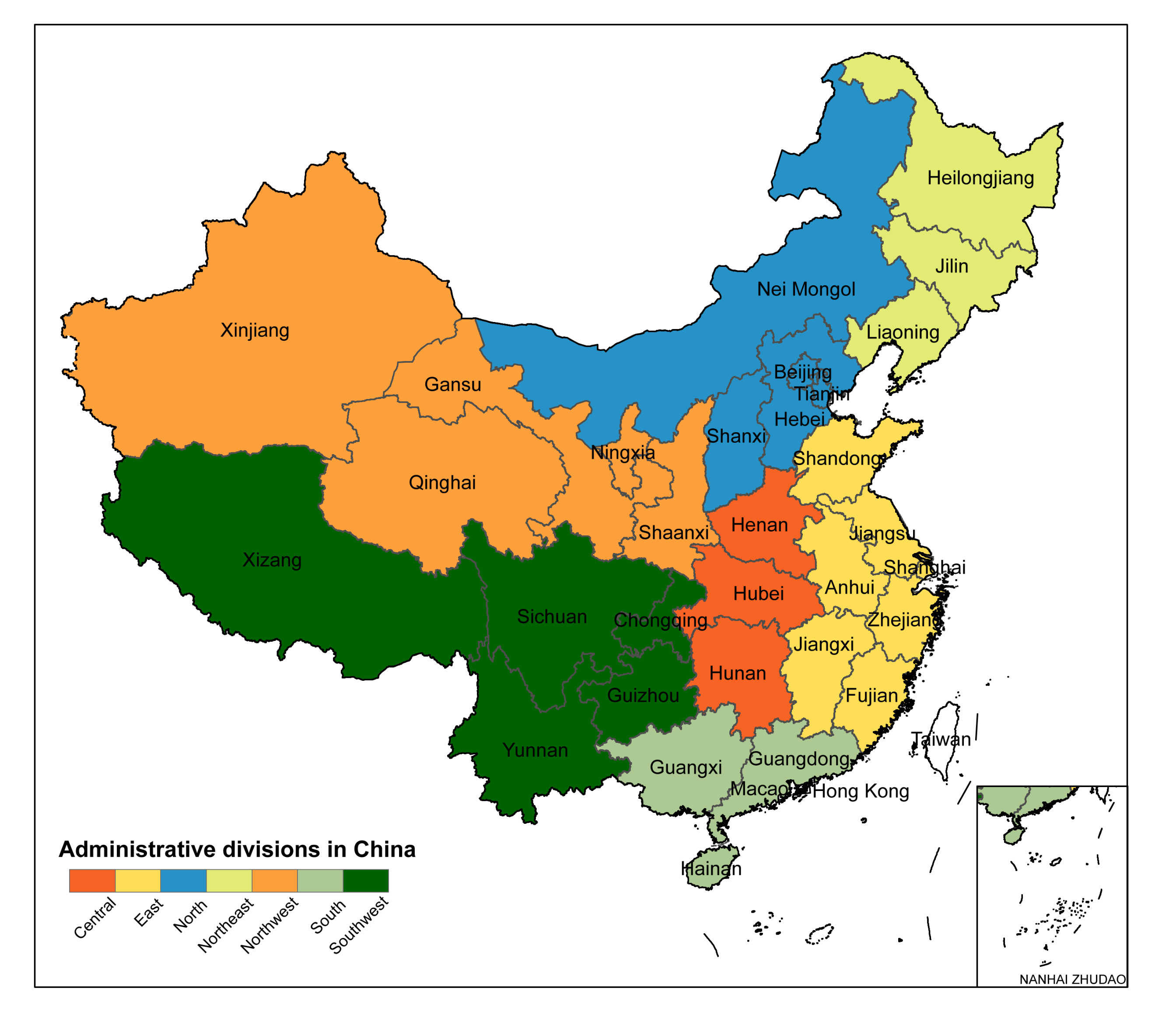


**Figure S11.** Administrative divisions in the mainland of China. Data from the Hong Kong, Macao, and Taiwan are not available in this study.





**Figure S12.** Observed versus simulated annual N_2_O fluxes from grasslands and forests in China.

**Table S1.** Comparison of China’s N_2_O emissions in this study with NGHGIs.

| **Sectors** | **Sources in this study** | **N_2_O emissions in this study (Gg N_2_O yr^-1^)** | | | | | **Sources in NGHGIs** | **N_2_O emissions in NGHGIs (Gg N_2_O yr^-1^)** | | | | |
| --- | --- | --- | --- | --- | --- | --- | --- | --- | --- | --- | --- | --- |
|  |  | **1994** | **2005** | **2010** | **2012** | **2014** |  | **1994** | **2005^b^** | **2010** | **2012** | **2014** |
| Energy | Electricity generation | 57.5 | 136.1 | 203.2 | 223.2 | 229.1 | Energy industries | 50.0 | 261.3 | 165.0 | 89.0 | 223.0 |
|  | Heat plants |  |  |  |  |  |  |  |  |  |  |  |
|  | Petroleum refining |  |  |  |  |  |  |  |  |  |  |  |
|  | Manufacture of solid fuels |  |  |  |  |  |  |  |  |  |  |  |
|  | Other energy industries |  |  |  |  |  |  |  |  |  |  |  |
|  | Manufacturing industries  and construction | 24.7 | 41.3 | 45.5 | 48.2 | 43.9 | Manufacturing Industries | ˗ |  | 53.0 | 52.0 | 65.0 |
|  | Transport | 4.2 | 11.4 | 17.1 | 20.3 | 20.4 | Transport | ˗ |  | 20.0 | 22.0 | 21.0 |
|  | Residential | 6.9 | 6.8 | 7.0 | 7.7 | 6.9 | Other sectors | ˗ |  | 7.0 | 7.0 | 7.0 |
|  | Agriculture/forestry/fishing/fish farms |  |  |  |  |  |  |  |  |  |  |  |
|  | Hon-specified | 1.0 | 0.9 | 2.0 | 0.8 | 0.9 | Others | ˗ |  | 63.0 | 55.0 | 51.0 |
|  | Fugitive emissions from fuels | 0.3 | 0.1 | 0.1 | 0.1 | 0.1 |  |  |  |  |  |  |
|  | **Subtotal** | **95^a^** | **197** | **275** | **300** | **301** | **Subtotal** | **50** | **261** | **308** | **224** | **367** |
| Industry | Nitric acid production | ˗ | 76.1 | 168.1 | 202.7 | 291.2 | Chemical industry | 15.0 | 106.5 | 200.0 | 255.0 | 311.0 |
|  | Adipic acid production |  |  |  |  |  |  |  |  |  |  |  |
|  | **Subtotal** |  | **76** | **168** | **203** | **291** |  | **15** | **106** | **200** | **255** | **311** |
| Agriculture | Manure left on pasture and manure management | 114.7 | 131.6 | 91.4 | 92.4 | 94.4 | Manure management | 786.0 | 1151.6 | 236.0 | 249.0 | 233.0 |
|  | Fertilizer application in cropland | 627.2 | 792.9 | 854.2 | 894.9 | 894.0 | Agricultural soils |  |  | 911.0 | 1218.0 | 930.0 |
|  | Nitrogen mineralization |  |  |  |  |  |  |  |  |  |  |  |
|  | Manure application in cropland |  |  |  |  |  |  |  |  |  |  |  |
|  | Crop residue |  |  |  |  |  |  |  |  |  |  |  |
|  | Fertilizer and manure application in pasture |  |  |  |  |  |  |  |  |  |  |  |
|  | Nitrogen deposition |  |  |  |  |  |  |  |  |  |  |  |
|  | Nitrogen leaching/runoff |  |  |  |  |  |  |  |  |  |  |  |
|  | Biomass burning | 3.6 | 4.0 | 4.5 | 4.8 | 4.9 | Field burning of agricultural residues |  |  | 7.0 | 8.0 | 7.0 |
|  | **Subtotal** | **745** | **928** | **950** | **992** | **993** |  | **786** | **1152** | **1154** | **1475** | **1170** |
| Waste | Biological treatment of solid waste and waste incineration | ˗ | 1.6 | 2.0 | 2.0 | 4.1 | Solid waste disposal on land & waste incineration | ˗ | 93.5 | 5.0 | 8.0 | 9.0 |
|  | Wastewater treatment and discharge | 70.1 | 89.7 | 102.1 | 105.7 | 109.5 | Wastewater handling |  |  | 96.0 | 97.0 | 110.0 |
|  | **Subtotal** | **70** | **91** | **104** | **108** | **114** | **Subtotal** |  | **94** | **101** | **105** | **119** |
| **Total** |  | **910** | **1293** | **1497** | **1603** | **1699** | **Total** | **850** | **1613** | **1764** | **2059** | **1967** |

^a^ Due to lack of N_2_O emission from energy sector in 1994, therefore this table uses values in 1995 here;

^b^ N_2_O emissions in 2005 from the NGHGIs are provided from the retrospective calculation in the Third National Communications on Climate Change.

**Table S2.** Comparison of EF_3_ in IPCC 2019 and IPCC 2006.

| **EF** | **Conditions** | | **Value** | |
| --- | --- | --- | --- | --- |
|  |  |  | IPCC 2019 | IPCC 2006 |
| EF_3_  (kg N_2_O-N kg^-1^ N) | Manure left on pasture | Cattle, swine, poultry | 0.004 | 0.02 |
|  |  | Other animals | 0.003 | 0.01 |
|  | Manure management | Lagoon | 0 | 0 |
|  |  | Liquid/Slurry | 0.005 | 0.005 |
|  |  | Solid storage | 0.01 | 0.005 |
|  |  | Dry lot | 0.02 | 0.02 |
|  |  | Pit | 0.002 | 0.002 |
|  |  | Digester | 0.0006 | 0 |
|  |  | Burned for fuel | 0.001 | 0.001 |
|  |  | Poultry manure with litter | 0.001 | 0.001 |
|  |  | Daily spread | 0 | 0 |

**Table S3.** Comparison of annual N excretion rate of livestock (*N_rate_*), mass of livestock (*T_AM_*), and fraction of manure in management systems (*P*) in IPCC 2019 and IPCC 2006.

| **Livestock** | **IPCC version** | **N_rate_ _(T)_ ^a^** | **T_AM(T)_^b^** | **P_(i, j)_^c^** | | | | | | | | | | |  |
| --- | --- | --- | --- | --- | --- | --- | --- | --- | --- | --- | --- | --- | --- | --- | --- |
|  |  |  |  | Pasture  /Range  /Paddock | | Lagoon | Liquid  /Slurry | Solid storage | Dry lot | | Pit | Digester | Burned  for fuel | Poultry manure  with litter | Daily spread |
| Dairy cattle | 2019 | 0.44 | 386 | | 0.38 | 0 | 0.01 | 0.21 | | 0.29 | 0 | 0 | 0.11 | 0 | 0 |
|  | 2006 | 0.47 | 350 | | 0.20 | 0.04 | 0.38 | 0 | | 0 | 0 | 0.02 | 0.07 | 0 | 0.29 |
| Other cattle | 2019 | 0.38 | 299 | | 0.36 | 0 | 0 | 0.29 | | 0.28 | 0 | 0 | 0.07 | 0 | 0 |
|  | 2006 | 0.34 | 319 | | 0.50 | 0 | 0 | 0 | | 0.46 | 0 | 0 | 0.02 | 0 | 0.02 |
| Goat | 2019 | 0.34 | 24 | | 0.50 | 0 | 0 | 0.5 | | 0 | 0 | 0 | 0 | 0 | 0 |
|  | 2006 | 1.37 | 30 | | - | - | - | - | | - | - | - | - | - | - |
| Sheep | 2019 | 0.32 | 31 | | 0.8 | 0 | 0 | 0.17 | | 0.03 | 0 | 0 | 0 | 0 | 0 |
|  | 2006 | 1.17 | 28 | | - | - | - | - | | - | - | - | - | - | - |
| Horse | 2019 | 0.46 | 238 | | 0.5 | 0 | 0 | 0.5 | | 0 | 0 | 0 | 0 | 0 | 0 |
|  | 2006 | 0.46 | 238 | | - | - | - | - | | - | - | - | - | - | - |
| Mule | 2019 | 0.46 | 130 | | 0.5 | 0 | 0 | 0.5 | | 0 | 0 | 0 | 0 | 0 | 0 |
|  | 2006 | 0.46 | 130 | | - | - | - | - | | - | - | - | - | - | - |
| Asses | 2019 | 0.46 | 130 | | 0.5 | 0 | 0 | 0.5 | | 0 | 0 | 0 | 0 | 0 | 0 |
|  | 2006 | 0.46 | 130 | | - | - | - | - | | - | - | - | - | - | - |
| Camel | 2019 | 0.46 | 217 | | 0.5 | 0 | 0 | 0.5 | | 0 | 0 | 0 | 0 | 0 | 0 |
|  | 2006 | 0.46 | 217 | | - | - | - | - | | - | - | - | - | - | - |
| Swine | 2019 | 0.61 | 58 | | 0 | 0.35 | 0.21 | 0 | | 0.02 | 0.35 | 0.07 | 0 | 0 | 0 |
|  | 2006 | 0.42 | 28 | | 0 | 0 | 0.4 | 0 | | 0.54 | 0 | 0.07 | 0 | 0 | 0 |
| Poultry | 2019 | 1.1 | 1.2 | | 0 | 0 | 0 | 0 | | 0 | 0 | 0 | 0 | 1 | 0 |
|  | 2006 | 0.82 | - | | - | - | - | - | | - | - | - | - | - | - |

^a^ N_rate(T)_: N excretion rate of livestock T, kg N (1000 kg animal mass)^-1^ day^-1^;

^b^ T_AM(T)_: mass of livestock T, kg animal^-1^;

^c^ P_(i, j)_: fraction of manure in the *j*th management system accounting for total manure of the *i*th livestock;

The uncertainties of N_rate(T)_, T_AM(T)_, and P_(i, j)_ were assigned as 50% due to the absence of data.

**Table S4.** Emission factors (EFs) applied in this study for estimating anthropogenic N_2_O emissions.

| **EF** | **Conditions** | | **Value** | | **Uncertainty (%)** | | **Ref.** |
| --- | --- | --- | --- | --- | --- | --- | --- |
| EF_1_ (kg N_2_O-N kg^-1^ N input) for agricultural synthetic fertilizer and manure application, N deposition, and N mineralization |  | | Upland | Paddy | Upland | Paddy | ^[2]^ |
|  | Region 1 (Xinjiang, Qinghai, Gansu, Ningxia, Shaanxi, Shanxi, Inner Mongolia, Tibet) | | 0.0065 | 0.0052 | 8.18 | 2.80 |  |
|  | Region 2 (Heilongjiang, Jilin, Liaoning) | | 0.0149 | 0.0161 | 14.45 | 19.30 |  |
|  | Region 3 (Beijing, Tianjin, Hebei, Henan, Shandong) | | 0.0079 | 0.0060 | 11.13 | 9.23 |  |
|  | Region 4 (Jiangsu, Anhui, Shanghai, Zhejiang, Jiangxi, Hubei, Hunan, Sichuan, Chongqing) | | 0.0157 | 0.0114 | 23.72 | 13.17 |  |
|  | Region 5 (Guangdong, Guangxi, Hainan, Fujian) | | 0.0086 | 0.0059 | 21.25 | 14.09 |  |
|  | Region 6 (Yunnan, Guizhou) | | 0.0093 | 0.0059 | 10.04 | 10.00 |  |
| EF_3_ (kg N_2_O-N kg^-1^ N excreted) for livestock excretion and manure management | Manure left on pasture | Cattle, swine, poultry | 0.004 | | 29.43 | | ^[3]^ |
|  |  | Other animals | 0.003 | | 25.00 | |  |
|  | Manure management | Lagoon | 0 | | 50^a^ | | ^[3]^ |
|  |  | Liquid/Slurry | 0.005 | | 21.81 | |  |
|  |  | Solid storage | 0.01 | | 50^a^ | |  |
|  |  | Dry lot | 0.02 | | 9.65 | |  |
|  |  | Pit | 0.002 | | 50^a^ | |  |
|  |  | Digester | 0.0006 | | 50^a^ | |  |
|  |  | Burned for fuel | 0.001 | | 50^a^ | |  |
|  |  | Poultry manure with litter | 0.001 | | 50^a^ | |  |
| EF_burn_ (kg N_2_O kg^-1^ dry matter burnt) for biomass burning | Agricultural residues | | 0.00007 | | 33.99 | | ^[3]^ |
| EF_5_ (kg N_2_O-N (kg N leaching and runoff)^-1^) for leaching and run off | Rivers | | 0.0022 | | 9.82 | | ^[2]^ |
|  | Estuary | | 0.0029 | | 2.17 | |  |
|  | Groundwater | | 0.0014 | | 14.79 | |  |
| EF_fuel_ (kg N_2_O TJ^-1^) for fuels | Crude oil | | 0.6 | | ^b^ | | ^[13]^ |
|  | Natural gas liquids | | 0.6 | |  |  |  |
|  | Gasoline | | 0.6 | |  |  |  |
|  | Kerosene | | 0.6 | |  |  |  |
|  | Gas/diesel oil | | 0.6 | |  |  |  |
|  | Fuel oil | | 0.6 | |  |  |  |
|  | Liquefied petroleum gases | | 0.1 | |  |  |  |
|  | Naphtha | | 0.6 | |  |  |  |
|  | Bitumen | | 0.6 | |  |  |  |
|  | Lubricants | | 0.6 | |  |  |  |
|  | Refinery gas | | 0.1 | |  |  |  |
|  | Paraffin waxes | | 0.6 | |  |  |  |
|  | Other petroleum products | | 0.6 | |  |  |  |
|  | Anthracite | | 1.5 | |  |  |  |
|  | Coking coal | | 1.5 | |  |  |  |
|  | Other bituminous coal | | 1.5 | |  |  |  |
|  | Lignite | | 1.5 | |  |  |  |
|  | Coke oven coke / lignite coke | | 1.5 | |  |  |  |
|  | Gas works gas | | 0.1 | |  |  |  |
|  | Coke oven gas | | 0.1 | |  |  |  |
|  | Blast furnace gas | | 0.1 | |  |  |  |
|  | Natural gas (dry) | | 0.1 | |  |  |  |
| EF_fug_ (kg N_2_O Gg^-1^) for fugitive emissions | Crude oil | | 7.60E-07 | |  | | ^[13]^ |
|  | Natural gas (dry) | | 2.50E-08 | |  |  |  |
| EF_ci_ (kg N_2_O t^-1^) for chemical industry | Nitric acid production | | 10 | | 15 | | ^[5]^ |
|  | Adipic acid production | | 300 | | 10 | |  |
| EF_solid_ (kg N_2_O kg^-1^ waste treated) for biological treatment of solid waste | Residual waste | | 0.0006 | | 85 | | ^[13]^ |
|  | Household food waste | | 0.00024 | | 85 | |  |
| EF_inc_ (kg N_2_O Gg^-1^ wet waste) for waste incineration | Waste incineration | | 50 | | 85 |  | ^[13]^ |
| EF_wt_ (kg N_2_O-N kg N^-1^) for wastewater treatment and discharge | Wastewater treatment and discharge | | 0.005 | | 400 |  | ^[13]^ |

^a^ The uncertainty was assigned as 50% due to the absence of data;

^b^ The uncertainty of EFs for each fuel in each use was listed in Table S15.

**Table S5.** Design of simulation experiments.

| **Model experiment** | **Abbr.** | **Period of drivers used** | | |
| --- | --- | --- | --- | --- |
|  |  | **CO_2_^a^** | **N_dep_^b^** | **CLIM^c^** |
| all variables constant | S0 | 1980 | 1980 | 1980 |
| only CO_2_ varying | S1 | 1980**–**2020 | 1980 | 1980 |
| CO_2_ and N_dep_ varying | S2 | 1980**–**2020 | 1980**–**2020 | 1980 |
| CO_2_, N_dep_ and CLIM varying | S3 | 1980**–**2020 | 1980**–**2020 | 1980**–**2020 |

^a^ CO_2_ indicates atmospheric CO_2_ concentration;

^b^ N_dep_ indicates atmospheric N deposition;

^c^ CLIM indicates climate change.

**Table S6**. Activity data not included in the national or provincial statistical system.

| **Sector** | **Source** | **Dataset** | **Ratio (%)^a^** |
| --- | --- | --- | --- |
| Agriculture | Manure management | Fraction of manure in each management system accounting for total manure of each livestock | 4.3 |
|  | Crop residue and biomass burning | Fraction of above-ground straw remaining in cropland and fraction of above-ground residues burnt for each crop type in each province | 3.4 |
| Industry | Adipic acid production | Production and emissions abatement | 13.8 |
|  | Nitric acid production | Production and emissions abatement | 1.9 |
| Energy | Transport | Fraction of fuels for each mode of transportation | 1.0 |
| Waste | Wastewater treatment and discharge | Technologies of wastewater treatment and discharge | 5.3 |
|  | Biological treatment of solid waste and waste incineration | Fraction of residual waste and household food waste | 0.2 |

^a^ Ratio indicated the N_2_O emission percentage of the corresponding category to the total N_2_O emission, which were calculated by the mean emission from 2010 to 2020.

**Table S7.** Sectors and categories included in Full-scale Annual N_2_O Dataset (FAN).

| **Sector** | **Code** | **Source** | **Resolution** | **Period** | **IPCC codes^a^** |
| --- | --- | --- | --- | --- | --- |
| Natural emissions | N1 | Forest | 0.25°×0.25° | 1980**–**2020 |  |
|  | N2 | Grassland | 0.25°×0.25° | 1980**–**2020 |  |
| Energy | E1 | Electricity generation | Province | 1985, 1995**–**2020 | 1.A.1.a.i |
|  | E2 | Heat plants | Province | 1985, 1995**–**2020 | 1.A.1.a.iii |
|  | E3 | Petroleum refining | Province | 1985, 1995**–**2020 | 1.A.1.b |
|  | E4 | Manufacture of solid fuels | Province | 1985, 1995**–**2020 | 1.A.1.c.i |
|  | E5 | Other energy industries | Province | 1985, 1995**–**2020 | 1.A.1.c.ii |
|  | E6 | Manufacturing industries and construction | Province | 1985, 1995**–**2020 | 1.A.2 |
|  | E7 | Transport | Province | 1985, 1995**–**2020 | 1.A.3 |
|  | E8 | Residential | Province | 1985, 1995**–**2020 | 1.A.4.b |
|  | E9 | Agriculture/forestry/fishing/fish farms | Province | 1985, 1995**–**2020 | 1.A.4.c |
|  | E10 | Hon-specified | Province | 1985, 1995**–**2020 | 1.A.5 |
|  | E11 | Fugitive emissions from fuels | Province | 1985, 1995**–**2020 | 1.B.2 |
| Industry | I1 | Nitric acid production | Province | 2002**–**2020 | 2.B.2 |
|  | I2 | Adipic acid production | Province | 2002**–**2020 | 2.B.3 |
| Agriculture | A1 | Fertilizer application in cropland | Province | 1980**–**2020 | 3.C.4 |
|  | A2 | Nitrogen mineralization | Province | 1980**–**2020 | 3.C.4 |
|  | A3 | Manure left on pasture and manure management | Province | 1980**–**2020 | 3.A.2 |
|  | A4 | Manure application in cropland | Province | 1980**–**2020 | 3.C.4 |
|  | A5 | Crop residue | Province | 1980**–**2020 | 3.C.4 |
|  | A6 | Nitrogen deposition | Province | 1980**–**2020 | 3.C.5 |
|  | A7 | Fertilizer and manure application in pasture | Province | 1980**–**2020 | 3.C.4 |
|  | A8 | Nitrogen leaching/runoff | Province | 1980**–**2020 | 3.C.5 |
|  | A9 | Biomass burning | Province | 1980**–**2020 | 3.C.1.b |
| Waste | W1 | Wastewater treatment and discharge | Province | 1981**–**2019 | 4.D |
|  | W2 | Biological treatment of solid waste and waste incineration | Province | 2002**–**2019 | 4.B & 4.C.1 |

^a^ IPCC codes mean the corresponding codes of each category in the 2019 Refinement to the 2006 IPCC Guidelines on National Greenhouse Gas Inventories.

**Table S8**. Comparison of data and methods used in this study with that in current main existing inventories.

| **Name** | **Sector** | **Period** | **Spatial resolution** | **Ref.** |
| --- | --- | --- | --- | --- |
| FAN v2020 | Energy, industry, agriculture, waste, natural source | 1980**–**2020 | Province and 25 km × 25 km (natural source) | This study |
| NGHGIs | Energy, industry, agriculture, waste | 1994, 2005, 2010, 2012, and 2014 | Country | ^[14-18]^ |
| EDGAR v7.0 | Energy, industry, agriculture, waste | 1980**–**2020 | 0.1° × 0.1° | ^[19]^ |
| GAINS | Energy, industry, agriculture, waste | 1990**–**2020  (every 5 years) | Province | ^[20]^ |
| FAOSTAT | Agriculture | 1980**–**2019 | Country | ^[21, 22]^ |

**Table S9.** Values of parameters applied in this study for estimating N_2_O emissions from crop residues and biomass burning ^[3]^.

| **Crop type** | **DRY^a^** | **R_AG_^b^** | **N_AG_^c^** | **RS^d^** | **N_BG_^e^** |
| --- | --- | --- | --- | --- | --- |
| Rice | 0.89 | 1.4 | 0.007 | 0.16 | 0.009 |
| Wheat | 0.89 | 1.3 | 0.006 | 0.26 | 0.009 |
| Maize | 0.87 | 1 | 0.006 | 0.22 | 0.007 |
| Soybean | 0.91 | 2.1 | 0.008 | 0.19 | 0.008 |
| Potato | 0.22 | 0.4 | 0.019 | 0.20 | 0.014 |
| Other grain | 0.85 | 1 | 0.008 | 0.22 | 0.009 |
| Peanut | 0.94 | 1 | 0.016 | 0.22 | 0.009 |
| Rapeseed | 0.85 | 1 | 0.008 | 0.22 | 0.009 |
| Sugar beet | 0.90 | 0.3 | 0.015 | 0.8 | 0.012 |
| Sugarcane | 0.80 ^[23]^ | 0.17 ^[24]^ | 0.015^f^ | 0.25 ^[25]^ | 0.012^f^ |

^a^ DRY: dry matter fraction of the harvest grain of crop;

^b^ R_AG_: dry matter ratio of above-ground residue to yield;

^c^ N_AG_: N content of above-ground residues, kg N kg ^-1^;

^d^ RS: ratio of below-ground biomass to above-ground biomass, kg kg ^-1^;

^e^ N_BG_: N content of below-ground residues, kg N kg ^-1^;

^f^ The values were set to the same as sugar beet;

The uncertainties of DRY, R_AG_, N_AG_, RS, and N_BG_ were set 20% following Zhou et al. ^[2]^.

**Table S10.** Values of P_Re_ and P_burn_ applied in this study for different provinces.

| **Province** | **P_Re_^a^** | **P_burn_^b^** | **Uncertainty of P_burn_ (%) ^[2]^** | **Province** | **P_Re_^a^** | **P_burn_^b^** | **Uncertainty of P_burn_ (%) ^[2]^** |
| --- | --- | --- | --- | --- | --- | --- | --- |
| Beijing | 0.60 ^[26]^ | 0.10 ^[27]^ | 12.50 | Hubei | 0.38 ^[28]^ | 0.20 ^[28]^ | 20.00 |
| Tianjin | 0.20 ^[26]^ | 0.17 ^[27]^ | 14.29 | Hunan | 0.71 ^[28]^ | 0.12 ^[28]^ | 20.00 |
| Hebei | 0.47 ^[28]^ | 0.08 ^[28]^ | 20.00 | Guangdong | 0.41 ^[28]^ | 0.08 ^[28]^ | 14.29 |
| Shanxi | 0.56 ^[28]^ | 0.04 ^[28]^ | 20.00 | Guangxi | 0.23 ^[29]^ | 0.23 ^[27]^ | 33.33 |
| Inner Mongolia | 0.14 ^[30]^ | 0.25 ^[27]^ | 100.00 | Hainan | 0.38 ^[28]^ | 0.25 ^[28]^ | 20.00 |
| Liaoning | 0.31 ^[28]^ | 0.01 ^[28]^ | 20.00 | Chongqing | 0.28 ^[31]^ | 0.12 ^[27]^ | 33.33 |
| Jilin | 0.04 ^[32]^ | 0.26 ^[27]^ | 14.29 | Sichuan | 0.14 ^[28]^ | 0.03 ^[28]^ | 33.33 |
| Heilongjiang | 0.35 ^[28]^ | 0.03 ^[28]^ | 14.29 | Guizhou | 0.15 ^[28]^ | 0.30 ^[28]^ | 33.33 |
| Shanghai | 0.48 ^[28]^ | 0.08 ^[28]^ | 12.50 | Yunnan | 0.31 ^[33]^ | 0.10 ^[27]^ | 33.33 |
| Jiangsu | 0.32 ^[28]^ | 0.07 ^[28]^ | 14.29 | Tibet | 0.14 ^[28]^ | 0.15 ^[27]^ | 100.00 |
| Zhejiang | 0.24 ^[28]^ | 0.12 ^[28]^ | 14.29 | Shaanxi | 0.32 ^[28]^ | 0.06 ^[28]^ | 20.00 |
| Anhui | 0.30 ^[28]^ | 0.15 ^[28]^ | 20.00 | Gansu | 0.27 ^[28]^ | 0.02 ^[28]^ | 33.33 |
| Fujian | 0.36 ^[28]^ | 0.11 ^[28]^ | 14.29 | Qinghai | 0.14 ^[30]^ | 0.16 ^[27]^ | 100.00 |
| Jiangxi | 0.65 ^[28]^ | 0.02 ^[28]^ | 20.00 | Ningxia | 0.07 ^[28]^ | 0.08 ^[28]^ | 33.33 |
| Shandong | 0.24 ^[28]^ | 0.06 ^[28]^ | 20.00 | Xinjiang | 0.14 ^[30]^ | 0.14 ^[27]^ | 33.33 |
| Henan | 0.35 ^[28]^ | 0.05 ^[28]^ | 20.00 |  |  |  |  |

^a^ P_Re_: fraction of above-ground straw remaining in cropland, uncertainty of P_re_ was set 20% following Zhou et al. ^[2]^;

^b^ P_burn_: fraction of above-ground residues being burnt.

**Table S11.** The fraction of N loss by leaching and runoff to N applications on managed soils (F_leach_) ^[2]^.

| **Crop type** | **Regions** | **F_leach_ (%)** | **Uncertainty (%)** |
| --- | --- | --- | --- |
| Paddy | Region 1 (Xinjiang, Qinghai, Gansu, Ningxia, Shaanxi, Shanxi, Inner Mongolia, Tibet) | 8.27 | 6.89 |
|  | Region 2 (Heilongjiang, Jilin, Liaoning) | 3.46 | 14.94 |
|  | Region 3 (Beijing, Tianjin, Hebei, Henan, Shandong) | 4.18 | 3.04 |
|  | Region 4 (Jiangsu, Anhui, Shanghai, Zhejiang, Jiangxi, Hubei, Hunan, Sichuan, Chongqing) | 3.39 | 31.65 |
|  | Region 5 (Guangdong, Guangxi, Hainan, Fujian) | 1.36 | 55.88 |
|  | Region 6 (Yunnan, Guizhou) | 1.21 | 56.12 |
| Upland | Region 1 (Xinjiang, Qinghai, Gansu, Ningxia, Shaanxi, Shanxi, Inner Mongolia, Tibet) | 6.53 | 9.60 |
|  | Region 2 (Heilongjiang, Jilin, Liaoning) | 0.61 | 123.28 |
|  | Region 3 (Beijing, Tianjin, Hebei, Henan, Shandong) | 4.48 | 24.67 |
|  | Region 4 (Jiangsu, Anhui, Shanghai, Zhejiang, Jiangxi, Hubei, Hunan, Sichuan, Chongqing) | 8.74 | 14.27 |
|  | Region 5 (Guangdong, Guangxi, Hainan, Fujian) | 7.23 | 8.48 |
|  | Region 6 (Yunnan, Guizhou) | 1.60 | 59.88 |

**Table S12.** Values of conversion factors (CF) applied in this study for fuel types ^[13]^.

| **Fuel types** | **CF (TJ Gg^-1^)** |
| --- | --- |
| Crude oil | 42.3 |
| Natural gas liquids | 44.2 |
| Gasoline | 44.3 |
| Kerosene | 43.8 |
| Gas/diesel oil | 43 |
| Fuel oil | 40.4 |
| Liquefied petroleum gases | 47.3 |
| Naphtha | 44.5 |
| Bitumen | 40.2 |
| Lubricants | 40.2 |
| Refinery gas | 49.5 |
| Paraffin waxes | 40.2 |
| Other petroleum products | 40.2 |
| Anthracite | 26.7 |
| Coking coal | 28.2 |
| Other bituminous coal | 25.8 |
| Lignite | 11.9 |
| Coke oven coke / Lignite coke | 28.2 |
| Gas works gas | 38.7 |
| Coke oven gas | 38.7 |
| Blast furnace gas | 2.47 |
| Natural gas (dry) | 48 |

**Table S13.** Observed annual N_2_O emissions from Chinese natural ecosystems for model validation.

| **Site** | **Longitude** | **Latitude** | **Vegetation type** | **Year observed** | **N_2_O fluxes (kg N m^-2^ yr^-1^)** | **Ref.** |
| --- | --- | --- | --- | --- | --- | --- |
| Wudaoliang, Tibet | 93.0°E | 35.0°N | Alpine steppe | 2000 | 0.007 | ^[34]^ |
| Beijing | 116.5°E | 39.5°N | Temperate forest | 1997**–**1998 | 0.028 | ^[35]^ |
| Changbai Mountain | 127.0°E | 41.5°N | Alpine tundra | 1994**–**1995 | 0.028 | ^[36]^ |
| Inner Mongolia | 116.0°E | 43.5°N | Temperate steppe | 1995 | 0.027 | ^[36]^ |
| Inner Mongolia | 116.5°E | 43.5°N | Temperate steppe | 1998 | 0.037 | ^[37]^ |
| Haibei, Qinghai | 101.5°E | 37.5°N | Alpine meadow | 2003 | 0.023 | ^[7]^ |
|  |  |  |  | 2004 | 0.016 |  |
|  |  |  |  | 2005 | 0.092 |  |
|  | 101.5°E | 38.0°N | Alpine shrub | 2003 | 0.07 |  |
|  |  |  |  | 2004 | 0.051 |  |
| Ruoergai, Sichuan | 102.5°E | 33.0°N | Alpine swamp | 2004 | 0.03 |  |
|  |  |  |  | 2005 | 0.031 |  |
| Fukang, Xinjiang | 87.5°E | 44.5°N | Temperate desert | 2004 | 0.031 |  |
|  |  |  |  | 2005 | 0.005 |  |
| Heshan, Guangdong | 113.0°E | 22.5°N | Subtropical forest | 2004 | 0.16 |  |
|  |  |  |  | 2005 | 0.226 |  |
| Xishuang banna, Yunnan | 102.0°E | 22.0°N | Tropical forest | 2003 | 0.122 |  |
|  |  |  |  | 2004 | 0.084 |  |
|  |  |  |  | 2005 | 0.105 |  |
| Dinghushan, Guangdong | 112.5°E | 23.0°N | Tropical forest | 2003 | 0.151 |  |
|  |  |  |  | 2004 | 0.139 |  |
|  |  |  |  | 2005 | 0.206 |  |
| Namco, Tibet | 90.5°E | 30.5°N | Alpine steppe | 2009 | 0.001 | ^[38]^ |

**Table S14.** Uncertainties of activity data.

| **Sector** | **Activity data** | | **Uncertainty (%)** | **Ref.** |
| --- | --- | --- | --- | --- |
| Agriculture | Fertilizer application |  | 9.2 | ^[2]^ |
|  | Manure application |  | 20^a^ |  |
|  | Nitrogen mineralization | | 20^a^ |  |
|  | Nitrogen deposition |  | 20^a^ |  |
|  | Crop production | Rice | 1.3 |  |
|  |  | Wheat | 0.5 |  |
|  |  | Maize | 0.9 |  |
|  |  | Other grains | 7.88 |  |
|  |  | Soybean | 1.1 |  |
|  |  | Potato | 29 |  |
|  |  | Sugarcane | 20 |  |
|  |  | Sugar beet | 20 |  |
|  |  | Peanut | 20 |  |
|  |  | Rapeseed | 0.5 |  |
|  | Livestock number | Dairy cattle | 5.6 |  |
|  |  | Other cattle | 5.6 |  |
|  |  | Goat | 20^a^ |  |
|  |  | Sheep | 7.4 |  |
|  |  | Horse | 1.7 |  |
|  |  | Mule | 20^a^ |  |
|  |  | Asses | 3.1 |  |
|  |  | Camel | 2.7 |  |
|  |  | Swine | 3.8 |  |
|  |  | Poultry | 5.2 |  |
| Energy | Energy industries | Liquid | 3 | ^[13]^ |
|  |  | Solid | 0.9 |  |
|  |  | Gaseous | 0.9 |  |
|  | Manufacturing industries and construction | Liquid | 2 |  |
|  |  | Solid | 1.7 |  |
|  |  | Gaseous | 1.6 |  |
|  | Road transportation | Diesel oil | 1 |  |
|  |  | Gaseous | 3 |  |
|  | Other sectors | Liquid | 6.5 |  |
|  |  | Solid | 18 |  |
|  |  | Gaseous | 7 |  |
|  | Other energy | Liquid | 15 |  |
|  |  | Solid | 20 |  |
|  |  | Gaseous | 20 |  |
|  | Oil and Natural gas and other emissions from energy production | | 90 |  |
| Industry | Nitric acid production | | 3 | ^[13]^ |
|  | Adipic acid production | | 3 |  |
| Waste | Wastewater treatment and discharge | | 9 | ^[13]^ |
|  | Biological treatment of solid waste and waste incineration | | 16 |  |

^a^ The uncertainty was assigned as 20% due to the absence of data.

**Table S15.** Uncertainties of EFs in energy sector.

| **Source** | **Condition** | **Uncertainty (%)** | **Ref.** |
| --- | --- | --- | --- |
| Energy industries | Liquid | 40 | ^[13]^ |
|  | Solid | 55 |  |
|  | Gaseous | 50 |  |
| Manufacturing industries and construction | Liquid | 45 |  |
|  | Solid | 50 |  |
|  | Gaseous | 45 |  |
| Road transportation | Diesel oil | 140 |  |
|  | Gaseous | 150 |  |
| Other sectors | Liquid | 40 |  |
|  | Solid | 55 |  |
|  | Gaseous | 40 |  |
| Other energy | Liquid | 50 |  |
|  | Solid | 60 |  |
|  | Gaseous | 60 |  |
| Oil and natural gas and other emissions from energy production |  | 60 |  |

**References**

1. Tian H, Bian Z, Shi H *et al.* HaNi: A Historical dataset of Anthropogenic Nitrogen Inputs to the terrestrial biosphere (1860-2019). 2022.

2. Zhou F, Shang Z, Ciais P *et al.* A new high-resolution N_2_O emission inventory for China in 2008. *Environ Sci Technol*. 2014; **48**(15): 8538-8547.

3. IPCC. 2019 Refinement to the 2006 IPCC Guidelines for National Greenhouse Gas Inventories. IPCC, Switzerland, 2019.

4. Land-Use Harmonization 2. <https://luh.umd.edu/data.shtml> (Accessed on 06 April 2023).

5. IPCC. Good practice guidance and uncertainty management in national greenhouse gas inventories. 2001.

6. Ma M, Song C, Fang H *et al.* Development of a process-based N_2_O emission model for natural forest and grassland ecosystems. *J Adv Model Earth Syst*. 2022; **14**(3): e2021MS002460.

7. Xu R, Wang Y, Wang Y *et al.* Estimating N_2_O emissions from soils under natural vegetation in China. *Plant Soil*. 2018; **434**(1-2): 271-287.

8. Sitch S, Smith B, Prentice I *et al.* Evaluation of ecosystem dynamics, plant geography and terrestrial carbon cycling in the LPJ dynamic global vegetation model. *Global Change Biol*. 2003; **9**(2): 161-185.

9. Yuan W, Liu D, Dong W *et al.* Multiyear precipitation reduction strongly decreases carbon uptake over northern China. *J Geophys Res: Biogeosci*. 2014; **119**(5): 881-896.

10. Shangguan W, Dai Y, Duan Q *et al.* A global soil data set for earth system modeling. *J Adv Model Earth Syst*. 2014; **6**(1): 249-263.

11. Yang J, Huang X. The 30 m annual land cover dataset and its dynamics in China from 1990 to 2019. *Earth Syst Sci Data*. 2021; **13**(8): 3907-3925.

12. Yuan W, Liu S, Liang S *et al.* Estimations of evapotranspiration and water balance with uncertainty over the Yukon River Basin. *Water Resour Manage*. 2012; **26**: 2147-2157.

13. IPCC. 2006 IPCC guidelines for national greenhouse gas inventories. IPCC, Switzerland, 2006.

14. NDRC. The People’s Republic of China Initial National Communication on Climate Change (in Chinese). <https://tnc.ccchina.org.cn/archiver/NCCCcn/UpFile/Files/Htmleditor/202007/20200723151547985.pdf> (Accessed on 06 April 2023).

15. NDRC. The People’s Republic of China Second National Communication on Climate Change (in Chinese). <https://tnc.ccchina.org.cn/archiver/NCCCcn/UpFile/Files/Htmleditor/202007/20200723151855183.pdf> (Accessed on 06 April 2023).

16. NDRC. The People’s Republic of China First Biennial Update Report on Climate Change. <https://www.ccchina.org.cn/archiver/ccchinacn/UpFile/Files/Default/20170124155928346053.pdf> (Accessed on 06 April 2023).

17. NDRC. The People’s Republic of China Second Biennial Update Report on Climate Change. <https://tnc.ccchina.org.cn/archiver/NCCCcn/UpFile/Files/Htmleditor/202007/20200723155226725.pdf> (Accessed on 06 April 2023).

18. NDRC. The People’s Republic of China Third National Communication on Climate Change (in Chinese). <https://tnc.ccchina.org.cn/archiver/NCCCcn/UpFile/Files/Htmleditor/202007/20200723152332694.pdf> (Accessed on 06 April 2023).

19. Crippa M, Solazzo E, Huang G *et al.* High resolution temporal profiles in the Emissions Database for Global Atmospheric Research. *Sci Data*. 2020; **7**(1): 1-17.

20. Winiwarter W, Höglund-Isaksson L, Klimont Z *et al.* Technical opportunities to reduce global anthropogenic emissions of nitrous oxide. *Environ Res Lett*. 2018; **13**(1): 014011.

21. FAO. The State of Food and Agriculture: Social protection and agriculture: breaking the cycle of rural poverty. Rome, Italy, 2015.

22. FAO. The State of Food Security and Nutrition in the World: Transforming food systems for affordable healthy diets. Rome, Italy, 2020.

23. Xie G, Wang X, Han D *et al.* Harvest index and residue factor of non-cereal crops in China (in Chinese). *J China Agric Univ*. 2011; **16**(1): 9-17.

24. Leal MRLV, Galdos MV, Scarpare FV *et al.* Sugarcane straw availability, quality, recovery and energy use: A literature review. *Biomass Bioenergy*. 2013; **53**: 11-19.

25. Zhang B, Zhou S, Yang G *et al.* Bionass and root characters of intergeneric hybrid Erianthus arundinaceus× Saccharum spontaneum and its progeny (in Chinese). *J China Agric Univ*. 2016; **21**(4): 18-25.

26. Fang F, Li X, Shi Z *et al.* Analysis on distribution and use structure of crop straw resources in Huang-Huai-Hai Plain of China (in Chinese). *Trans Chin Soc Agric Eng*. 2015; **31**(2).

27. Zhou Y, Xing X, Lang J *et al.* A comprehensive biomass burning emission inventory with high spatial and temporal resolution in China. *Atmospheric Chemistry and Physics*. 2017; **17**(4): 2839-2864.

28. Gao X, Ma W, Ma C *et al.* Analysis on the Current Status of Utilization of Crop Straw in China (in Chinese). *J Huazhong Agric Univ*. 2002; **21**: 242-247.

29. Li Q. Study on the comprehensive utilization of straw in Guangxi (in Chinese), *Master Thesis*. Guangxi University, College of Agriculture, 2013.

30. Bao J, Yu J, Feng Z *et al.* Situation of distribution and utilization of crop straw resources in seven western provinces, China (in Chinese). *Chin J Appl Ecol*. 2014; **25**(1): 181-187.

31. Li M, He W, Li Z *et al.* Study on the distribution characteristics and utilization of crop straw in chongqing (in Chinese). *South China Agric*. 2013; **7**(9): 32-34.

32. Liu P, Na W, Wang X *et al.* Analysis on evaluation and energy utilization of main crop stalk resource in Jilin Province (in Chinese). *J Jilin Agric Sci*. 2010; **35**(5): 58-64.

33. National crop straw resources investigation and evaluation report (in Chinese). Information Office of the Ministry of Agriculture, 2011.

34. Pei Z. Carbon dynamics in the alpine grassland ecosystem on the Tibetan Plateau — a case study of Wudaoliang, Qinghai province, *PhD Thesis*. Institute of Geographic Sciences and Natural Resources Research, 2003.

35. Sun X, Xu H. Emission flux of nitrous oxide from forest soils in Beijing (in Chinese). *Sci Silvae Sin*. 2001; **37**: 57-63.

36. Chen G, Huang B, Xu H *et al.* Nitrous oxide emissions from terrestrial ecosystems in China. *Chemosphere Global Change Sci*. 2000; **2**(3-4): 373-378.

37. Xu R, Wang Y, Zheng X *et al.* A comparison between measured and modeled N_2_O emissions from Inner Mongolian semi-arid grassland. *Plant Soil*. 2003; **255**: 513-528.

38. Wei D, Xu R, Wang Y *et al.* Responses of CO_2_, CH_4_ and N_2_O fluxes to livestock exclosure in an alpine steppe on the Tibetan Plateau, China. *Plant Soil*. 2012; **359**: 45-55.
